# Generation of the functionally enhanced proximal tubular cells from human iPSC-derived kidney organoids

**DOI:** 10.1038/s12276-026-01821-0

**Published:** 2026-09-03

**Authors:** Sua Kim, Sun Ah Nam, Jin Won Kim, Jawoon Yi, Jin Ju Woo, Minji Lee, Jeongeun Lee, Tae-Hyung Kim, Hyunglae Kim, Hyung Wook Kim, Jiwhan Park, Yong Kyun Kim

**Affiliations:** 1 Cell Death Disease Research Center, College of Medicine, The Catholic University of Korea, Seoul, Korea; 2 Rgenorgano Biotechnology, Seoul, Korea; 3 School of Life Sciences, Gwangju Institute of Science and Technology (GIST), Gwangju, Korea; 4 Department of Intelligent Precision Healthcare Convergence, Institute for Cross-disciplinary Studies (ICS), Sungkyunkwan University (SKKU), Suwon, Korea; 5 Department of Internal Medicine, The Catholic University of Korea, St Vincent’s Hospital, Seoul, Korea

**Keywords:** Stem-cell differentiation, Regeneration

## Abstract

Human renal proximal tubular epithelial cells (RPPTCs) are crucial for exploring the molecular and cellular mechanisms underlying the pathophysiology of kidney injuries. In this study, we generated the functionally enhanced kidney organoid-derived proximal tubule cells (FEKOPTCs) from human induced pluripotent stem cells. Heparan sulfate proteoglycans and transforming growth factor-β receptor inhibitor enhanced the functionality of PTCs isolated from the kidney organoids of which the vascularization and maturation were improved by heparan sulfate proteoglycans and vascular endothelial growth factor. FEKOPTCs showed significantly enhanced epithelial barrier function, polarization state, and transporter function, compared with primary human RPTECs, which led to improve the function of drug uptake. Transcriptomic characterization of FEKOPTCs revealed high similarity to PTCs of native human adult kidney. FEKOPTCs-on-chip are more sensitive predictors than RPTECs-on-chip for nephrotoxicity testing. By using CRISPR−Cas9 genetic mutation of alpha-galactosidase A, the causative gene of Fabry disease, alpha-galactosidase A-mutant FEKOPTCs, recapitulated the functional phenotype of Fabry disease. When FEKOPTCs were transplanted into the kidney parenchyma of NOD-SCID mice, they reassembled and regenerated proximal tubular structures with intact lumens and functional transporters. Taken together, our results suggest that FEKOPTCs are functionally enhanced PTCs and can be applied for nephrotoxicity testing, disease modeling, and regenerative medicine.

## Enhanced kidney organoids improve proximal tubule cell function

Kidney proximal tubular cells (PTCs) are vital for maintaining kidney function; however, current in vitro models fall short in predicting nephrotoxicity. This study addresses this by developing functionally enhanced kidney organoid-derived PTCs using human induced pluripotent stem cells. Researchers optimized protocols to mature kidney organoids with heparan sulfate proteoglycans and vascular endothelial growth factor, enhancing vascularization and maturation. The functionally enhanced kidney organoid-derived PTCs exhibited improved barrier function, polarization, and transporter activity compared with existing models. These cells demonstrated high transcriptomic similarity to native human PTCs, making them effective for nephrotoxicity testing and disease modeling, including Fabry disease. Transplantation into mice showed regenerative potential, forming proximal tubule-like structures. This advancement in generating mature PTCs from organoids holds promise for drug testing, disease modeling, and regenerative therapies, with future directions focussing on broader applications in kidney-related research and treatment. This summary was initially drafted using artificial intelligence, then revised and fact-checked by the author.

## Introduction

Kidney proximal tubular cells (PTCs) have a critical role in maintaining life by regulating the water balance and acid–base balance and maintaining electrolyte homeostasis. Currently, isolated primary human renal proximal tubular cells (RPTECs) or human kidney 2 (HK-2) cells, an immortalized PTC line from a normal adult human kidney, are used as in vitro models of PTCs for screening compounds to determine their nephrotoxicity and for investigating the mechanistic and functional effects of drug exposure^1,2^. However, these in vitro models often fail to accurately predict nephrotoxicity because they cannot recapitulate relevant aspects of kidney physiology^3,4^. From a clinical application perspective, PTCs become useful as they become more functionally and morphologically mature.

Human kidney organoids derived from human induced pluripotent stem cells (iPSCs) are an attractive source of PTCs because they contain nephron components that recapitulate human kidney development^5^. Previous studies reported that PTCs isolated from kidney organoids have an epithelial barrier function and transporters and can be used for nephrotoxicity tests^6–8^.

However, the immaturity of kidney organoids remains a concern^9,10^. Direct comparisons with kidney tissue in vivo reveal that kidney organoids formed in vitro remain relatively immature^5^. This immaturity could limit the clinical use of PTCs isolated from human kidney organoids^9,10^.

We previously reported that kidney decellularized extracellular matrix (dECM) enhances the vascularization and maturation of human kidney organoids by providing a microenvironment suitable for differentiation^11^. Kidney dECM has many matrisome protein components, growth factors, and cytokines, making it a functional material with a native kidney environment^11^.

Despite its beneficial effects on the maturation of kidney organoids, kidney dECM has several limitations^12^. To better mimic the complex environment of human kidney, human native kidney-derived dECM is ideal^12^. However, the availability of human native kidneys can be restricted due to ethical issues. In addition, differences in the source, decellularization methods, and preparation processes for kidney dECM can produce differences in its composition that affects consistency between batches.

Therefore, for this study, we devised a strategy to address the limitations of kidney dECM while maintaining its beneficial functions for kidney organoid maturation. We selected the components in kidney dECM that we expected to play the most important role in the maturation of kidney organoids, and then optimized the protocols.

Among the core components of kidney dECM, we focussed on heparan sulfate proteoglycans (HSPGs) and vascular endothelial growth factor (VEGF). HSPGs constitute up to 30% of the matrisome proteins in kidney dECM. They confer a negative charge to basement membranes and link laminin and collagen IV networks^13,14^. HSPGs can sequester soluble growth factors important to cell viability and transduce signals to maintain endothelial cell stasis, and they are angiogenic factors that contribute to differentiation and thereby have a significant role in cell growth and maturation^15,16^.

VEGF enhanced vascularization of kidney organoids^11^. We previously reported that the vascularization and maturation of kidney organoids are abundantly enhanced, especially when VEGF was added to kidney dECM^11^. In this study, we demonstrated the mechanism why the combination of VEGF and HSPG, core proteins in kidney dECM, has a synergistic effect on the vascularization and maturation of kidney organoids. On the basis of those findings, we developed the protocol to differentiate “matured and vascularized kidney organoids” with an appropriate combination of HSPGs and VEGF.

We isolated PTCs from the matured and vascularized kidney organoids generated with this protocol and optimized the protocols to improve the functionality of the isolated PTCs and generated functionally enhanced kidney organoid-derived proximal tubule cells (FEKOPTCs). The FEKOPTCs showed significantly improved barrier function, polarization state, and transporter function compared with RPTECs, which led to improved drug uptake. Transcriptomic characterization of FEKOPTCs revealed high similarity to PTCs of native human adult kidney. Unlike kidney organoids, FEKOPTCs can be cryopreserved and serially passaged without loss of identity.

We tested the clinical application of FEKPOTCs. FEKOPTCs-on-chip show epithelial polarization and membrane transporter activity, which makes them effective in drug-induced nephrotoxicity assays. The transcellular transport of substrates and PTC maturation were more improved in microfluidic conditions than in static conditions. We found that FEKOPTCs-on-chip are more sensitive predictors of nephrotoxicity than RPTECs.

We also found that FEKOPTCs can phenocopy kidney disease. Fabry disease is an inherited lysosomal storage disease caused by an alpha-galactosidase A (GLA) mutation. GLA-mutant KOPTCs were generated using CRISPR–Cas9 genetic editing of GLA in human iPSCs. The GLA-mutant FEKOPTCs recapitulated the phenotype and main pathogenesis of Fabry kidney disease, the accumulation of globotriaosylceramide (Gb3), and that accumulation was recovered with recombinant enzyme replacement therapy (ERT), the primary therapy for Fabry disease. These findings support the usefulness of KOPTCs as screening tools for the development of novel therapies for Fabry disease.

We also tested the regenerative potential of FEKPOTCs. When we transplanted FEKOPTCs into the kidneys of NOD-SCID mice, they reassembled in the kidneys of host mice and regenerated proximal tubular structures with intact lumens and transporters.

Our study highlights that FEKOPTCs are functionally enhanced PTCs similar to PTCs of native human adult kidney and can be applied for nephrotoxicity testing, disease modeling, and regenerative medicine.

## Materials and methods

### Generation of human iPSCs from peripheral blood mononuclear cells (PBMCs)

PBMCs were collected into heparinized tubes from a healthy female who provided written informed consent. PBMCs were isolated with Ficoll-Paque^TM^ PLUS (catalogue number, 17-1440-03, GE Healthcare, Little Chalfont, Buckinghamshire, UK) and maintained in 24-well culture plates with StemSpan medium (9805, STEMCELL Technologies, Vancouver, British Columbia, Canada) for 4–5 days. The human induced pluripotent stem cells were generated through overexpression using a Sendai virus reprogramming kit (A16518, Life Technologies, Inc., Carlsbad, CA, USA) with the PBMCs. The next day, we transferred the cells to 24-well plates coated with Geltrex, centrifuged them at 2,250 rpm for 10 min, and replaced the medium with TeSR-E8 medium (5940, STEMCELL Technologies). This study was approved by the Institutional Review Board of St Vincent’s Hospital, Suwon (IRB No. VC21TISI0008).

### Differentiation of the vascularized and matured kidney organoids from human iPSCs

**Fig. 1:**
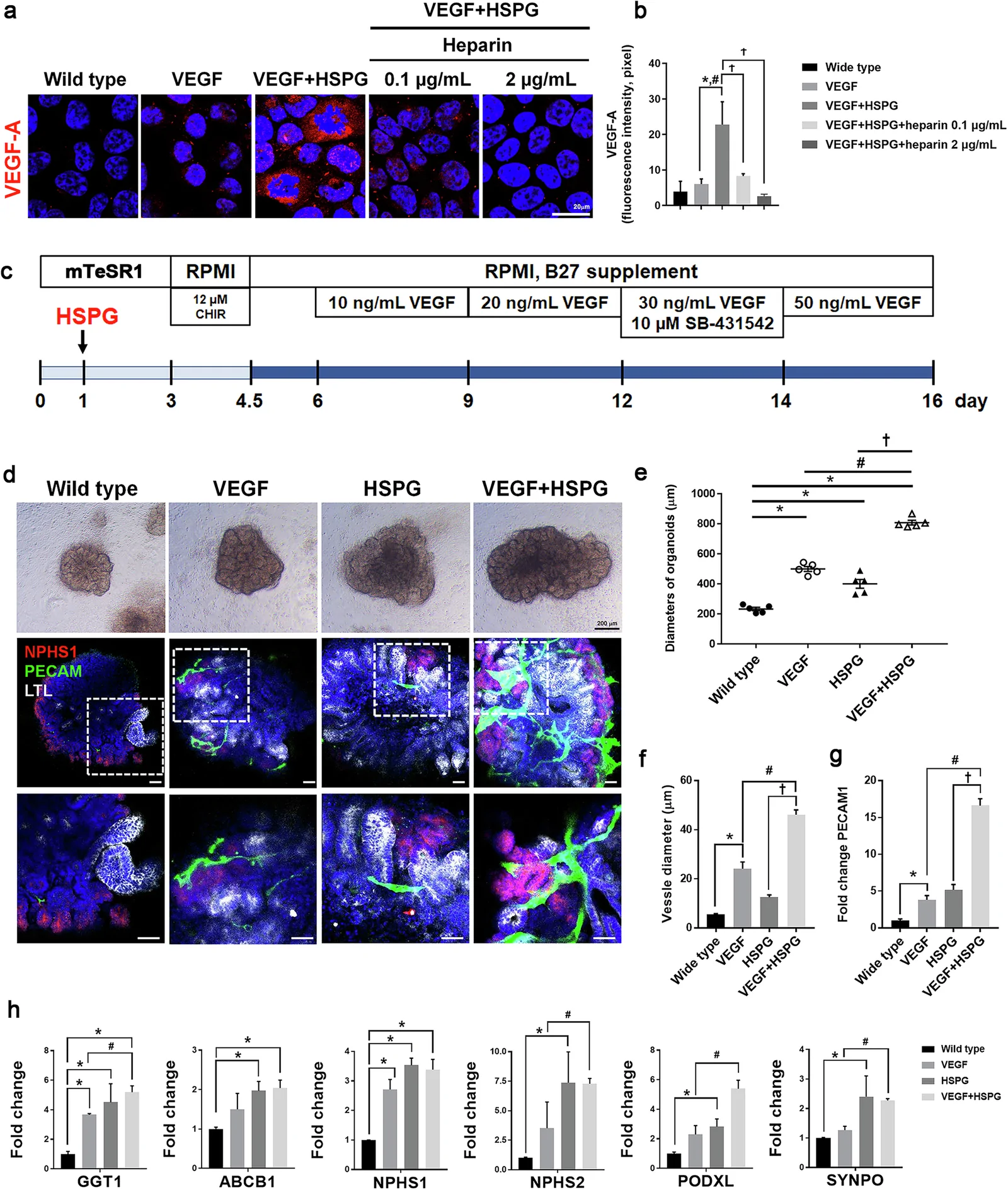
Differentiation of vascularized and mature kidney organoids from human iPSCs. **a**,**b** Immunofluorescent staining and qRT-PCR analysis of vascular endothelial growth factor (VEGF)-A by treatment with VEGF or VEGF + heparan sulfate proteoglycan (HSPG) in the absence or presence of heparin in HEK293T cells. **c** A protocol for the differentiation of vascularized and mature kidney organoids. **d** Representative bright field image and immunofluorescent staining of the morphology of kidney organoids cultured from each group. Immunofluorescent staining of LTL (proximal tubule), PECAM1 (vascular network), and NPHS1 (podocyte) in kidney organoids. Scale bar, 20 µm. **e** Quantification of diameters of kidney organoids among four groups (*n* = 5). **f** Quantification of the diameter of the kidney organoid vessels among four groups. **g** qRT-PCR analysis of PECAM1 in kidney organoids among four groups (*n* = 3). **h** qRT-PCR analysis of GGT1 (proximal tubule gene), ABCB1 (drug transporter genes), NPHS1, NPHS2, and PODXL, SYNPO (podocyte gene) (*n* = 3). Values are mean ± SEM. *, versus wild-type; #, versus VEGF; †, versus HSPG by the post hoc Mann–Whitney *U*-test.

The human iPSCs were passaged two times before seeding for differentiation. Kidney organoid differentiation was performed as previously described^17^. In brief, human iPSCs at a density of 15,000 cells/ml were plated in a 6-well plate coated with 1% Geltrex (Thermo Fisher Scientific), containing mTeSR1 medium (Stem Cell Technologies) + 10 µM Y27632 (LC Laboratories) (day 0). After 24 h, the human iPSCs were sandwiched between a lower layer of HSPG (Sigma-Aldrich, Catalog no. H4777) and an upper layer of Geltrex, with the addition of 1.5% Geltrex and 0.6 µg/ml HSPG. On day 3, CHIR was administered to the cells, which were then fed with an appropriate medium every 3 days, as described in Fig. 1c, to facilitate kidney organoid differentiation. VEGF was added on days 6, 9, 12, and 14 to enhance vascular development. Additionally, SB-431542 was added on day 12 to promote podocyte differentiation.

### Isolating PTCs from the kidney organoids

The kidney organoids were harvested after 15–17 days of induction and then dissociated using Accutase enzyme solution for 10 min at 37 °C. In brief, the samples were centrifuged at 200×*g* for 4 min, resuspended, and filtered through a 40 µm pluriStrainer sieve to eliminate any remaining cell aggregates. Biotinylated LTL, a well-established proximal tubule brush border marker, was added to the suspension at a dilution of 1:100 as the primary antibody. The supernatant was kept on ice for 30 min before 3 ml of magnetic-activated cell sorting (MACS) isolation buffer was added, followed by centrifugation at 200×*g* for 4 min. After we aspirated the supernatant, we washed the cells with another 3 ml of MACS isolation buffer and then centrifuged them again using the same parameters. After the supernatant was removed, the cells were resuspended in 90 µl of MACS isolation buffer, and 10 µl of streptavidin magnetic beads was incorporated into the sample, which was kept on ice for 30 min. Following this incubation, an additional 3 ml of MACS isolation buffer was added, and the sample was centrifuged at 200×*g* for another 4 min. The supernatant was then aspirated, and the cells were washed in 3 ml of MACS isolation buffer. A final centrifugation at 200×*g* for 4 min was performed, and the cells were resuspended in 5 ml of MACS isolation buffer. An MACS column (Miltenyi Biotec 130-042-401) was placed into an MACS magnet for MACS sorting. The magnetic column was removed from the magnet, placed into a new 15 ml conical tube, and designated as LTL-positive PTCs. LTL-positive PTCs were counted and seeded onto wells pretreated with laminin and HSPG. The media were changed every 24 h post-isolation to eliminate apoptotic cells.

### Generation of FEKOPTCs from mKOPTCs

The matured kidney organoids-derived proximal tubular cells (mKOPTCs) were cultured in 6-well culture plates precoated with laminin-511 and HSPG in DPBS. For passages 0 and 1, the mKOPTCs were cultured in DMEM/F12 Basal Media supplemented with 2 mM Glutamax (Gibco, no. 35050-061), 1% ITS (insulin-transferrin-Selenium, Gibco, no. 41400-045), 10 ng/ml human EGF (Sigma-Aldrich, no. E9644), 50 nM hydrocortisone (Sigma-Aldrich, no. H6909), 1 nM triiodothyronine (Sigma-Aldrich, no. T6397), 1% FBS, and 0.5% penicillin/streptomycin. Upon reaching passage 2, the passage was performed when the cells reached ~90% confluency by first washing the cells with DPBS without calcium and magnesium, treating with 0.05% trypsin/EDTA for 5 min, followed by quenching with culture medium, centrifuging at 440 g for 4 min, resuspending in culture media, and seeding each dish. Additionally, SB-431542 (10 nM, Selleckchem) was added as a transforming growth factor (TGF)-β/activin/NODAL pathway inhibitor. Cells were then fed every 2–3 days. From passage 5 onwards, cells were referred to as FEKOPTCs.

Primary human renal proximal tubule epithelial cells (RPTECs) from Lonza (no. CC-2553) were cultured in an REGM^TM^ Renal Epithelial Cell Growth Medium BulletKit^TM^ (Lonza, no. CC-3190). The HK-2 cells from ATCC were cultured in DMEM containing 10% FBS and 1% penicillin/streptomycin and were added to all media. All the cells were maintained in 5% CO_2_ incubator at 37 °C.

### Immunofluorescence microscopy

For the immunofluorescence analysis, organoids were fixed on day 18, unless specified otherwise. The fixation procedure involved adding an equal volume of PBS (Thermo Fisher Scientific) mixed with 8% paraformaldehyde (PFA) (Electron Microscopy Sciences) to the medium for 15 min, after which the samples were washed three times with PBS. Cells including FEKOPTCs and RPTECs were fixed in 4% PFA in PBS, pH 7.4, for 20 min at RT. After fixation, both the organoids and cells were washed twice in PBS and then permeabilized using 0.3% Triton X-100 in PBS at RT for 1 h. The fixed organoids and cells were blocked in 5% donkey serum (Millipore, Billerica, MA, USA) + 0.3% Triton-X-100/PBS, incubated overnight in 0.1% bovine serum albumin (Sigma-Aldrich, St Louis, MO, USA) + PBS with primary antibodies, washed, incubated with fluor-conjugated secondary antibodies (Invitrogen, Carlsbad, CA, USA), washed, and stained with DAPI or mounted in Vectashield H-1000. Images were acquired using a Zeiss LSM 700 confocal microscope (Carl Zeiss, Germany) and ZEN 3.1 software. The following primary antibodies were used: anti-LTL (Vector Labs, no. FL-1321), anti-NPHS1 (R&D Systems, no. AF4269), anti-acetylated tubulin (Sigma-Aldrich, no. 7451), PECAM1 (Abcam, no. ab9498), anti-ZO-1 (Thermo Fisher Scientific, no. 33-9100), anti-laminin (Sigma-Aldrich, no. L9393), anti-ECAD (Abcam, no. ab11512), anti-SGLT2 (Abcam, no. ab85626), anti-N-cadherin (Thermo Fisher Scientific, no. MA1-91128), anti-Gb3 (TCI chemicals, no. A2506), anti-OAT1 (Alpha Diagnostic, no. OAT11-A), and anti-OAT3 (Alpha Diagnostic, no. OAT31-A).

### Real-time quantitative PCR analysis

Kidney organoid samples and cells were harvested, and total RNA was isolated using Trizol reagent (Life Technologies, Inc.). Each sample was derived from three individual samples. RNA was quantified by spectrophotometry, and cDNA synthesis was performed using 2 μg of RNA and SuperScript^®^ III reverse transcriptase (Life Technologies, Inc.). For qPCR, 100 ng of cDNA served as a template for PCR amplification, which used Brilliant SYBR green QPCR master mix according to the manufacturer’s instructions (FastStart DNA Master SYBR Green I; Roche Molecular Biochemicals, Mannheim, Germany). Serial dilutions (1 ng/μl to 1 fg/μl) of cDNA were used as templates to generate a standard curve. Nested primers were used to amplify the standard and kidney cDNA samples (Supplementary Table 1). Standard and unknown samples were amplified in duplicate in 96-well plates. The thermal profile of the LightCycler^®^ instrument (Roche Molecular Biochemicals) was optimized using an initial denaturation for 5 min at 95 °C, followed by 45 amplification cycles each consisting of 10 s at 95 °C, 45 s at 60 °C, and 10 s at 72 °C. The comparative Ct method was used to determine the relative amounts of the target mRNA, expressed for each sample as a percentage of the GAPDH mRNA level. The Ct ratio was analyzed using LightCycler software (Version 4.05). Specificity was verified by post-run melting-curve analyses.

### Processing and analysis of RNA-seq

Total RNA was isolated using Trizol reagent (Invitrogen). RNA quality was assessed by a TapeStation4000 system (Agilent Technologies, Amstelveen, The Netherlands), and RNA quantification was performed using an Invitrogen Qubit 4 (Thermo Inc., DE, USA). Libraries were prepared from total RNA using a CORALL RNA-Seq V2 library prep kit (LEXOGEN, Inc., Austria). The isolation of mRNA was performed using a Poly(A) RNA selection kit (LEXOGEN, Inc.). The isolated mRNAs were used for cDNA synthesis and shearing, following the manufacturer’s instructions. Indexing was performed using Illumina indexes 1–12. The enrichment step was carried out using PCR. Subsequently, the libraries were checked using a TapeStation HS D1000 screen tape (Agilent Technologies) to evaluate the mean fragment size. Quantification was performed using a library quantification kit and a StepOne real-time PCR system (Life Technologies, Inc.). High-throughput sequencing was performed as paired-end 150 sequencing using a NovaSeq XP (Illumina, Inc., USA).

Raw RNA-sequencing (RNQ-seq) data were subjected to quality control and adapter trimming using Trin Galore (v.0.6.10), a wrapper tool around Cutadapt and FastQC. Trimmed reads were aligned to the Homo sapiens reference genome (GRCh38) using STAR aligner (v2.7.10a). The resulting BAM files were used to quantify gene-level expressing using featureCounts function from the Rsubread R package (v2.14.2). Gene annotation was based on the GTF file corresponding to the GRC38 reference genome. Only uniquely aligned reads overlapping exons were counted, and a count matrix was generated for downstream analysis.

### Differential expression analysis

Raw count matrices from Rsubread were analyzed in R using the edgeR package (v3.42.4). Lowly expressed genes were filtered using filterByExpr function, retaining only genes with sufficient counts for reliable statistical inference. The filtered data were normalized using the trimmed mean of M-values (TMM) method via calcNormFactors.

### Comparative analysis with single-cell reference data

Following normalization and filtering, transcriptomic profiles from the experimental dataset were compared against reference data to assess similarity with kidney PTCs.

Single-cell RNA-seq data from the Kidney Cell Atlas (https://www.kidneycellatlas.org/) were used as reference. Proximal tubule-related cell types were subset from the mature kidney dataset and aggregated into a pseudo-bulk expression profile using the AverageExpression function in the Seurat R package (v5.1.0), which computes the average normalized expression across all cells in each cluster. Pearson correlation analysis was then performed between the log-transformed normalized expression values (logCPM) of the experimental bulk RNA-seq samples and the pseudo-bulk PT profile to quantify transcriptomic similarity.

### Functional enrichment analysis

To assess the biological relevance of the selected genes, Gene Ontoloty (GO) enrichment analysis was performed using the enrichGO function from the clusterProfiler R package (v4.8.3). Gene symbols were converted to Entrez IDs using the bitr function and the org.Hs.eg.db annotation package.

Enrichment analysis was conducted for the ‘Biological Process’ category (ont = ‘BP’), with *P*-value cut-off of 0.05. Multiple testing correction was performed using the Benjamin–Hochberg method. Enriched GO terms were visualized using bar plot.

### Projection of bulk RNA-seq onto single-cell UMAP

To further evaluate transcriptomic similarity between FEKOPTC cells and native human PT cells, we projected FEKOPTC bulk RNA-seq profiles onto the UMAP embedding of a publicly available human kidney single-cell RNA-seq dataset (GSE131685).

UMAP coordinates and cell-type annotations were produced using the authors’ published processing pipeline from their GitHub repository (https://github.com/lessonskit/Single-cell-RNA-sequencing-of-human-kidney). The single-cell data were processed using the Seurat package (v.5.1.0), and dimension reduction steps (PCA, UMAP) were replicated as described in the original study.

Pearson correlation was calculated between log-normalized FEKOPTC expression and each single cell in the reference dataset using shared genes. The resulting correlation scores were used as weights to compute a correlation-weighted centroid, representing the projected position of the bulk sample in the reference UMAP space.

### Cellular deconvolution using BisqueRNA

To infer the cellular composition of FEKOPTC, we performed reference-based deconvolution using the BisqueRNA R package (v1.0.5). The single-cell reference dataset (GSE131685) was converted into an ExpressionSet object, and FEKOPTC expression data were normalized using log2(CPM + 1) transformation. The ReferenceBasedDecomposition function was executed with default parameters. Resulting cell-type proportion estimates were analyzed to determine the relative contributions of proximal tubule-related subtypes.

### Sequencing analysis

Quality control of the raw sequencing data was performed using FastQC^18^. Adapter and low-quality reads were removed using Fastp^19^. Then, the trimmed reads were mapped to the reference genome using STAR^20^. The quantification of reads was processed using Salmon^21^. The read counts were processed based on the TMM + CPM normalization method in the Python “conorm” package. Data mining and graphic visualization were performed using ExDEGA (Ebiogen Inc., Korea).

### Measurement of TEER

The intactness of the paracellular pathway in the FEKOPTC and RPTEC monolayers against small ions was monitored by measuring the TEER using an EVOM^TM^ apparatus (World Precision Instruments, Sarasota, FL, USA). For the measurement of TEER, FEKOPTCs and RPTECs were seeded onto Transwell filters (diameter 24 mm, pore size 0.4 μM, Corning, NY, USA). TEER was normalized to the area of the filter after removing the background resistance of a blank filter that did not have any cells seeded onto it and contained only culture medium. TEER was measured as Ω·cm^2^ and was zero when determined without cells.

### Assessment of paracellular permeability

To measure inulin leakage, Transwell™ filters were washed once with Hanks’ balanced salt solution (HBSS, Gibco) with 1% HEPES (Thermo Fisher Scientific). Then, 0.2 ml of 0.05 mg ml inulin-FITC (2 to 5 kDa, Sigma) in HBSS with 1% HEPES was added to the apical side, whereas 0.6 ml of HBSS with 1% HEPES was added to the basolateral side. RPTECs and FEKOPTCs were incubated at 37 °C for 90 min, and then duplicates of 200 μl were sampled from the basolateral compartment and transferred to black, flat-bottom 96-well plates (Greiner). Fluorescence was measured (*λ*_ex_ = 485 nm and *λ*_em_ = 530 nm) using a Fluorescence microplate reader (Gemini EM). Baseline fluorescence (HBSS with 1% HEPES) was subtracted from the measured values. Percent leakage was determined as (duplicate mean basolateral/duplicate mean apical) *100%.

### Assessment of MRP2/4 transport function

For measurements of selective excretion, RPTECs and FEKOPTCs were seeded onto Transwell filters until confluency was reached and then differentiated for 7 days. After differentiation, the leak tightness of the filters was assessed. The Transwell filters were washed once with HBSS containing 1% HEPES. Next, 0.2 ml of 10 µM carboxydichlorofluorescein diacetate (CDFDA, Sigma) with or without 20 µM MK571 (Sigma) in HBSS with 1% HEPES was added to the apical chamber. In the basolateral chamber, HBSS with 1% HEPES, with or without 20 µM MK571, was added. The cells were incubated at 37 °C for 45 min. Following incubation, duplicates of 100 µl were taken from the basolateral compartment and transferred to black, flat-bottomed 96-well plates (Greiner). Fluorescence was measured using a fluorescence microplate reader (Gemini EM) with excitation at 485 nm and emission at 530 nm. Baseline fluorescence, measured in HBSS with 1% HEPES, was subtracted from the recorded values. The percentage of leakage was calculated as (mean basolateral duplicate/mean apical duplicate) × 100%.

### Scanning electron microscopy (SEM) analysis

Confluent cell monolayers were washed twice with PBS, fixed in 4% PFA, and 2.5% glutaraldehyde with 0.1 M phosphate buffer overnight at 4°C. After washing in 0.1 M phosphate buffer, the samples were post-fixed in the same buffer containing 1% osmium tetroxide for 1 h at RT. Cells were dehydrated through a graded series of ethanol and finally hexamethyldisilazane for overnight and then left to air-dry. After samples were mounted on a Pt ion sputter coater (SMC12R-Plus; Semian, Inc., Daejeon, Republic of Korea), the cells were examined in an SEM (Regulus 8220, Hitachi, Ltd, Tokyo, Japan) at 2 kV.

### Transmission electron microscopy (TEM) analysis

Confluent cell monolayers were washed twice with PBS and fixed in 4% PFA and 2.5% glutaraldehyde with 0.1 M phosphate buffer overnight at 4 °C. After washing in 0.1 M phosphate buffer, the samples were post-fixed in the same buffer containing 1% osmium tetroxide for 1 h at RT. Next, the samples were dehydrated with a series of graded ethylalcohol solutions, exchanged through acetone, and embedded in Epon 812. Ultrathin sections of 100 nm thicknesses were prepared and observed under an electron microscope (HT7800, Hitachi, Ltd), contrasted with 1% uranyl acetate and lead citrate staining.

### Construction of FEKOPTCs-on-chip

Assembled microfluidic slide chambers were precoated with laminin-511 and HSPG in DPBS at 37 °C for 1 h. Then, RPTECs and FEKOPTECs were seeded into separate chambers in a suspension of 5 × 10^5^ cells per ml, with 30 µl of the cell suspension applied to the slide channel of each microslide. Then, we covered each chamber with the supplied lid and incubated it at 37 °C for 3 h to ensure that each reservoir contained 60 µl of cell-free fluid medium. After 3 h, we started the perfusion of complete medium using a dual-syringe pump (SyringeTWO, no. NE-4000, NewEra Pump Systems) at a flow rate of 1 µl/min. The RPTECs and KOPTCs typically reached confluency by day 10.

### Analyzing cell viability and nephrotoxicity with microfluidic systems

The viability of the cells was determined using a Live/Dead kit (CCK-8, DOJINDO Molecular Technologies, no. CK04-01). Dehydrogenases reduced the CCK-8 solution in cells to give an orange product (WST-8 formazan). After 1 h in the incubator, the absorbance at 450 nm was measured at the top-inlets and outlets using a microplate reader (Versa Max, Molecular Devices).

### Cisplatin exposure and lactate dehydrogenase (LDH) activity assay

To determine the toxic effects of cisplatin on FEKOPTCs, medium for static or fluidic devices was added on day 7 after seeding in the presence of 0, 0.1, 1, 10, 50, 100, or 200 µM cisplatin (Selleckchem, no. S1166). After incubation for 48 h, a medium sample from the outlet tubing was put into a falcon tube and used for the LDH activity assay. Next, the tubes were incubated with WST-8 to determine cell viability.

Following the manufacturer protocol, the LDH activity of the cisplatin-exposed samples was determined using an LDH activity assay kit (Invitrogen, no. C20300). Briefly, 10 μl of ultrapure water was added to the sample from the outlet tubing medium to determine the release of LDH into the cell culture medium (spontaneous LDH release), or 10 μl of 10× lysis buffer provided in a CyQUANT™ LDH cytotoxicity assay kit (Thermo Fisher Scientific) was added and incubated at 37 °C for 45 min to determine the total LDH content of the cells (maximum LDH release). In duplicate, 50 μl of each sample medium (spontaneous and maximum LDH activity controls) was added to a new 96-well plate. The reaction mixture provided in the CyQUANT™ LDH cytotoxicity assay kit was added to each well and mixed by tapping. The plates were incubated for 30 min, and then 50 μl of stop solution was added. Absorbance measurements were recorded on a BioTek Synergy HT microplate reader at 490 nm. The spontaneous and maximum LDH release control absorbance values were plotted after subtracting the background absorbance measurement. For this analysis, LDH activity was determined using the following formula:

$$\tfrac{{\rm{Cisplatin\; treated\; LDH\; activity}}-{\rm{Spontaneous}}\, \% \, \% \,{\rm{cyto\; activity}}}{{\rm{Maximum\; LDH\; activity}}-{\rm{Spontaneous\; LDH\; activity}}}\times 100={\rm{Normalized\; LDH\; release}}$$

### Generation of GLA-mutant FEKOPTCs from human iPSCs

The generation of GLA-mutant human iPSCs was described previously. To generate GLA (α-galactosidase A) knockout (KO) human iPSCs, an all-in-one CRISPR–Cas9-OFP plasmid targeting exon 1 of the human GLA gene (gRNA sequence: 5′-TTGGCAAGGACGCCTACCAT-3′) was constructed using the GeneArt CRISPR Nuclease Vector Kit (cat. no. A21174, Thermo Fisher Scientific, USA), which includes a vector encoding SpCas9 and OFP reporter. The oligo annealing and subcloning into the Cas9 nuclease reporter vector were performed according to the manufacturer’s instructions. For transfection, 1 × 10^6^ cells/ml iPSCs were electroporated with 10 µg of the constructed plasmid using the Nepa21 Transfection System (NEPAGENE) under the following optimized conditions: 125 V, 5 ms, and 2 pulse, following the manufacturer’s protocol for human pluripotent stem cells. Post-electroporation, cells were immediately seeded onto Geltrex-coated 6-well plates in mTeSR1 medium supplemented with 10 µM Y-27632 to enhance viability.

Next day of transfection, OFP-positive cells were sorted using FACSCanto II (BD Biosciences). Single OFP+ cells were sorted into 96-well plates containing mTeSR1 medium and maintained under feeder-free conditions until colonies expanded. A total of six OFP-positive clones were established. Genomic DNA was extracted using InstaGene Matrix (Bio-Rad, cat. no. 732-6030) and the CRISPR-targeted GLA region was PCR-amplified and sequenced by Sanger sequencing (Macrogen, Seoul, Korea). Among the six clones, two (clone 5 and clone 9) exhibited frameshift-inducing mutations at the target site. Clone 9 was subsequently used to generate GLA-mutant kidney organoids.

Clone 9 was identified as a GLA mutant because it was found to harbor an alteration at the genetic lesion targeted by the GLA-specific single guide RNA-mediated CRISPR–Cas9. It was used for western blot analyses. Next, the GLA-mutant kidney organoids were differentiated from GLA-mutant human iPSCs by the methods described earlier. mKOPTCs were isolated from GLA-mutant kidney organoids and cultured by the methods described earlier, and finally GLA-mutant FEKOPTCs were generated.

### Immunoblot analysis

We conducted immunoblotting of the GLA9 mutant–FEKOPTCs. Total protein was isolated from GLA9 mutant–FEKOPTCs using lysis buffer prepared by mixing T-PER tissue protein extraction reagent (Invitrogen, cat. no. 78510) with a protease and phosphatase inhibitor tablet (Fisher, cat. no. PIA32961), according to the manufacturer’s instructions. Protein concentration was measured using the Bradford assay reagent (Bio-Rad, Hercules, CA, USA). Equal amounts of the protein sample were separated by SDS–PAGE, and the proteins were transferred electrophoretically from unstained gels to polyvinylidene fluoride membranes (Bio-Rad). To detect GLA9 mutant FEKOPTCs, the samples were run on a NuPAGE^TM^ 10% tris-acetate protein gel (Invitrogen, EA03785), and a protein ladder (Invitrogen, cat. no. LC5699) was used in each gel to track 46-kDa proteins. After the transfer was completed, the membranes were blocked with skimmed milk in PBS-T (80 mM Na_2_HPO_4_, 20 mM NaH_2_PO_4_, 100 mM NaCl, 0.1% Tween-20, pH 7.5) for 1 h, and then the membranes were probed overnight at 4 °C with the following primary antibodies: rabbit polyclonal anti-GLA (Thermo Fisher Scientific, no. PA5-27349) and mouse monoclonal anti-β-actin (Sigma-Aldrich, no. A5441). The secondary antibodies were goat anti-rabbit (no. 111-035-003) and donkey anti-mouse IgG (no. 715-035-151) conjugated to horseradish peroxidase (Jackson ImmunoResearch, West Grove, PA, USA). The sites of antibody–antigen reactions were viewed using enhanced chemiluminescence (WEST-ZOL®Plus, Intron Biotechnology, Sungnam, Korea), and the band densities of the immunoblots were quantified by densitometry using a laser scanner and Quantity One software (Version 5.2, Bio-Rad Laboratory, Berkeley, CA, USA).

### Oil Red O staining

We stained cells in the GLA9 mutant–FEKOPTCs with Oil Red O to evaluate lipid droplet accumulation in the cytoplasm. GLA9 mutant–FEKOPTCs were fixed in 4% PFA (Electron Microscopy Services), and then the Oil Red O staining solution was applied to the fixed cells for 10–15 min, after which excess dye was rinsed off with distilled water. Counterstaining with hematoxylin was optionally conducted to visualize the cell nuclei. Microscopic examination revealed red-stained lipid droplets, indicating lipid accumulation within the cells.

### Mitochondrial staining and ROS detection

For mitochondrial staining, cells were washed once with PBS and then treated with 5 mM MitoTracker (Thermo Fisher Scientific, no. M7511) and 200 mM Hoechst (Thermo Fisher Scientific, no. 62249). After an incubation period of 1 h, a fluorescence image was obtained using a confocal microscope. MitoSOX red (Thermo Fisher Scientific, no. M36008) was used to measure superoxide anion levels. The GLA9 mutant–KOPTCs were treated with Hoechst 33342 (200 mM) for 1 h and MitoSOX red (5 mM) for 30 min at 37 °C in darkness. Afterward, the GLA-mutant–KOPTCs were washed with PBS, and fluorescence images were obtained using a confocal microscope.

### Measurement of intracellular calcium potential

To detect their intracellular calcium potential, the GLA9 mutant–FEKOPTCs were washed with PBS and treated with a Fluo-8 calcium assay kit (5 mM, AAT Bioquest) for 1 h at 37 °C in darkness. Then, the GLA9 mutant–FEKOPTCs were washed with PBS, and a *z*-stack fluorescence image was obtained using a fluorescence microscope.

### Transplantation of FEKOPTCs into the parenchyma of mouse kidney

The mice were anesthetized with isoflurane, and the dorsal flank was disinfected and incised to expose the kidney. To prevent bleeding during the procedure, the renal artery and vein were temporarily clamped using microvascular clamps, and a PE50 tube containing FEKOPTCs (2 × 10^6^ cells/ml) was directly injected into the mouse kidney. The delivery of the FEKOPTCs was facilitated by carefully blowing through the opposite end of the PE50 tube. Mice were euthanized 14 days post-transplantation. All surgical procedures, along with presurgical and postsurgical care for the animals, complied with the Laboratory Animals Welfare Act, the Guide for the Care and Use of Laboratory Animals, and the Guidelines and Policies for Rodent Survival Surgery set forth by the Institutional Animal Care and Use Committee at the School of Medicine, Catholic University of Korea (Approval no. CUMS-2024-0127-03).

### Statistical analysis

Data are presented as mean ± standard deviation. The Kruskal–Wallis non-parametric test was used to assess differences among the groups, and comparisons between control and treatment and between treatment and co-treatment were performed using the Mann–Whitney *U*-test (GraphPad Software, La Jolla, CA, USA). *P*-values <0.05 were considered statistically significant.

## Results

### Enhancement of the maturation and vascularization of human iPSCs-derived kidney organoids

To generate functionally enhanced and mature PTCs, it is critical to generate the matured and vascularized kidney organoids that serve as a cell source for the generation of PTCs. We previously demonstrated that kidney dECM promotes the maturation and vascularization of kidney organoids^11^. HSPG, a major component of kidney dECM, is a highly acidic polysaccharide found on the cell surface and in the extracellular matrix that attracts external ligands and facilitates their cellular entry^22^. In our previous study, the treatment of VEGF on kidney dECM accelerated the maturation and vascularization of kidney organoids more compared with when kidney dECM was treated alone^11^. Thus, we hypothesized that the HSPG attracts VEGF and facilitates its cellular entry, which may enhance the vascularization of kidney organoids^11^. Figure 1a,b shows that the combination treatment of HSPG and VEGF increased the expression of VEGF-A in the cytoplasm of HEK293T (human embryonic kidney-derived) cells compared with VEGF was treated alone. Moreover, heparin, which competes with cell-surface HSPG moieties^23^, caused a dose-dependent decrease in cytoplasmic VEGF-A expression, demonstrating the role of HSPG factors in VEGF intracellular entry (Fig.1a,b).

On the basis of these findings, we optimized the concentrations of HSPG and VEGF that most significantly promoted vascularization of kidney organoids (Supplementary Fig. 1a–c). We found that the gene expression of PECAM-1 increased in kidney organoids treated with HSPG at a concentration of 0.6 µg ml, but was not further increased at higher concentrations (Supplementary Fig. 1a). In addition, we determined the gene expression pattern of PECAM-1 in kidney organoids when HSPG was treated with VEGF (Supplementary Fig. 1b). When HSPG at a concentration of 0.3 µg ml was treated with VEGF, there was no difference in the expression of PECAM-1 compared with the kidney organoids treated with VEGF alone, whereas when HSPG at a concentration of 0.6 µg ml was treated with VEGF, PECAM-1 expression significantly increased. Therefore, for the differentiation of kidney organoids, we selected 0.6 µg ml as the concentration of HSPG (Supplementary Fig. 1b). We also selected 0.6 µg ml as the most appropriate concentration of HSPG for vascularization of kidney organoids. Next, we optimized the timing of HSPG treatment to enhance the gene expression of PECAM1. When HSPG was treated in the early stage during the period of differentiation, the gene expression of PECAM1 increased more significantly than when it was treated in the late stage (Supplementary Fig. 1c). Furthermore, the early-stage treatment of HSPG increased the gene expression of kidney cells more than late-stage treatment (Supplementary Fig. 1d–f).

On the basis of these experimental results, we developed 3D culture systems to differentiate human iPSCs into kidney organoids with the appropriate combination of HSPG and VEGF (Fig. 1c). In this protocol, each well of a 6-well culture plate was coated with diluted Geltrex, after which dissociated, undifferentiated human iPSCs were evenly placed. After 24 h, we added 0.6 µg ml HSPG with 1.5% Geltrex, sandwiching the human iPSCs. The sandwiched human iPSCs formed compact, ball-like colonies within 48 h and then developed internal cavities. On day 3, we treated the cells with CHIR 12 µM and fed with an appropriate medium every 2–3 days to promote kidney organoid differentiation. We added VEGF on days 6, 9, 12, and 14 and SB-431542 on day 12 (Fig. 1c).

To clarify the synergistic effect of HSPG and VEGF on the differentiation of kidney organoids, the experiments were performed with four groups: a group with kidney organoids generated using a Matrigel-based protocol (group 1), a group with a Matrigel-based protocol with VEGF (group 2), a group with a Matrigel-based protocol with HSPG (group 3), and group 4, a group with a Matrigel-based protocol with HSPG and VEGF.

Bright field microscopy revealed that the kidney organoids in group 4 had tubular structures, and they were larger than the kidney organoids in groups 1, 2, and 3 (Fig. 1d,e). Vascularization patterns were assessed in these groups. Confocal microscopy showed that vascularization of PECAM1+ endothelial cells was limited in group 1 and increased slightly in groups 2 and 3. Vascularization extensively increased and accelerated in group 4 (Fig. 1d–g). The diameters of the PECAM1-positive vascular structures were largest in group 4 (Fig. 1d,f). Real-time quantitative polymerase chain reaction showed that the gene expression of the vascular marker PECAM1 was upregulated in group 4 compared with their expression in groups 1, 2, and 3 (Fig. 1g).

Vascularization enhanced their maturation in kidney organoid differentiation as well as kidney development^24,25^. Thus, we determined the maturity and functional potential in these groups. The gene expression of drug transporter genes in PTCs (GGT1, ABCB1) and podocyte genes (NPHS1, NPHS2, PODXL, SYNPO) was most upregulated in group 4 among the groups (Fig. 1h).

When the kidney organoids were transplanted beneath the capsule of the kidneys of NOD-SCID mice, the kidney organoids in group 4 were more vascularized and expressed the genes of podocytes and PTCs compared with those in group 1 (Supplementary Fig. 2a–e).

Taken together, these findings suggest that the effect of HSPG and VEGF on vascularization and maturation of kidney organoids in group 4 may be the most suitable as a cell source for generating functionally enhanced and matured PTCs.

### Isolation of PTCs from kidney organoids

**Fig. 2:**
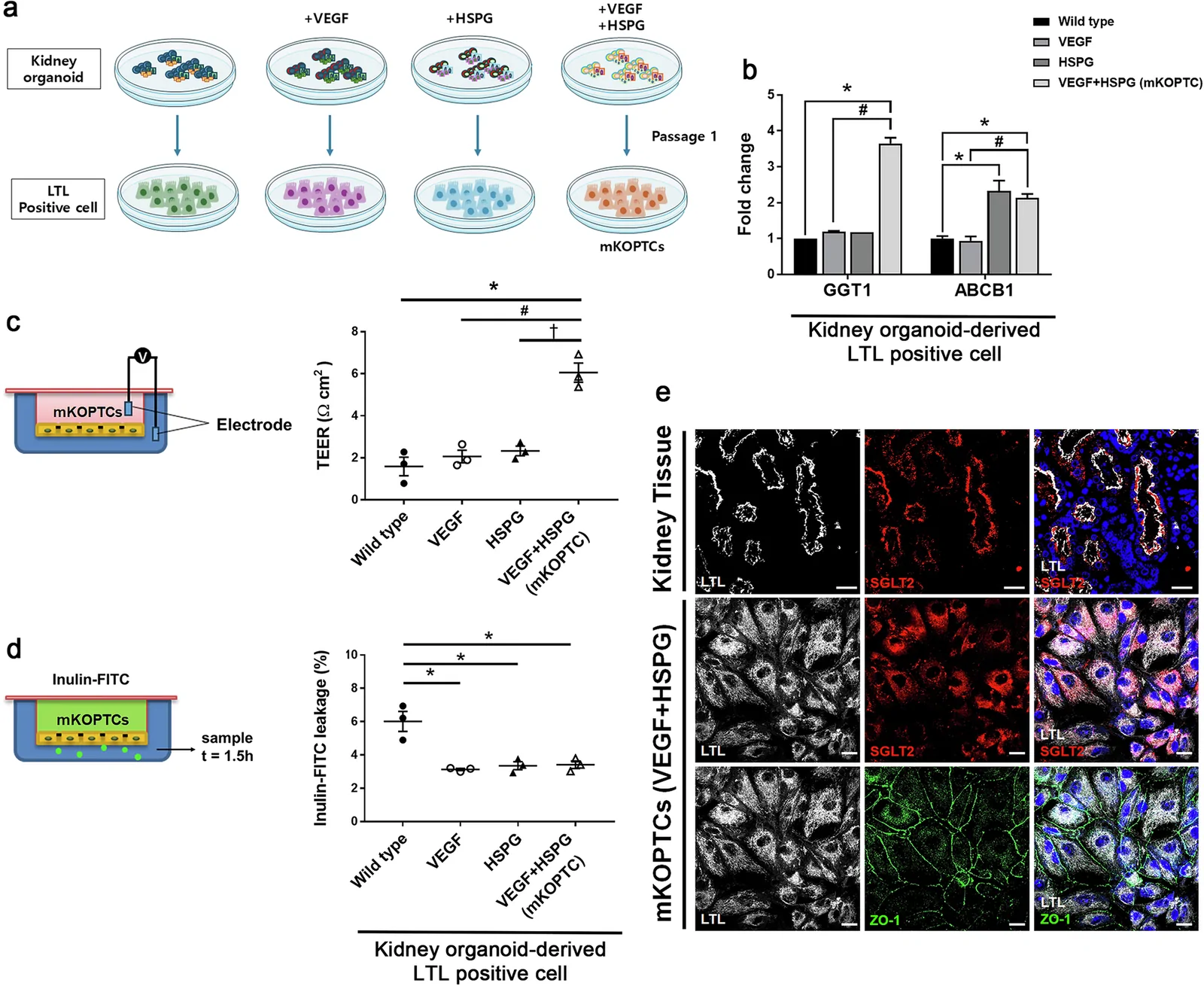
Enhanced functionality and maturity of mKOPTCs (matured kidney organoid-derived PTCs). **a** Schematic overview of process to compare characteristics among four groups of the isolated LTL-positive cells from the kidney organoids according to the differentiation protocols. **b** qRT-PCR analysis of GGT1 (proximal tubule gene) and ABCB1 (drug transporter genes) (*n* = 3). **c** Comparison of transepithelial electrical resistance (TEER) of the isolated LTL-positive cells from the kidney organoids differentiated among four groups. **d** Comparison of inulin-FITC leakage (*n* = 3, four technical replicates each, analyzed together). Values are mean ± SEM. *, versus wild-type; #, versus VEGF; †, versus HSPG by the post hoc Mann–Whitney *U*-test. **e** Representative confocal fluorescence images showing LTL (proximal tubule marker), SGLT2 (glucose transporter), and ZO-1 (tight junction protein) in kidney tissue and mKOPTC. Scale bar, 20 µm.

To validate that group 4 kidney organoids were the most suitable cell source for generating functionally enhanced and mature PTCs, we isolated LTL-positive cells, a marker of PTCs, from kidney organoids of each group by MACS and compared them (Fig. 2a). The expression of drug transporter genes (GGT1, ABCB1) was upregulated in the isolated LTL-positive cells from kidney organoids in group 4 (Fig. 2b).

The epithelial barrier function of PTCs is crucial for maintaining homeostasis in the kidney, and its disruption is a hallmark of numerous diseases and toxic effects. We assessed the epithelial barrier function with the measurement of transepithelial electrical resistance (TEER) and inulin-fluorescein isothiocyanate (FITC) leakage of the cells grown as monolayers on a Transwell system (Fig. 2c,d). TEER was highest in the isolated LTL-positive cells from kidney organoids in group 4 (Fig. 2c). The leakage of inulin-FITC in the isolated LTL-positive cells from kidney organoids in group 4 was lower than those of group 1 (Fig. 2d). These findings indicate that the isolated LTL-positive cells from kidney organoids in a group with a Matrigel-based protocol with HSPG and VEGF had better epithelial barrier function compared with those in other groups.

Furthermore, immunofluorescence analysis showed that the isolated LTL-positive cells from kidney organoids in a group 4 expressed the markers of PTCs, LTL, and SGLT2 as well as junctional protein (ZO-1), which is similar to in vivo kidney tissue (Fig. 2e).

Taken together, these data suggest that the isolated LTL-positive cells from kidney organoids differentiated by protocol based on HSPG and VEGF treatment (group 4), called mKOPTCs, are most suitable as a cell source for generating functionally enhanced and matured PTCs among groups.

### Generation of FEKOPTCs

Next, we developed a protocol to improve the maturity and functionality of mKOPTCs. The mKOPTCs were cultured in a well coated with HSPG, and the culture conditions were optimized. The gene expression of functional transporters of PTCs including SCL34A1, SGLT2, and ABCB1 was highest when the KOPTCs were cultured on an extracellular matrix composed of 0.66 µg/ml HSPG with a 2.5 µg/ml laminin-coated dish in DPBS media without calcium and magnesium (Supplementary Fig. 3).

**Fig. 3:**
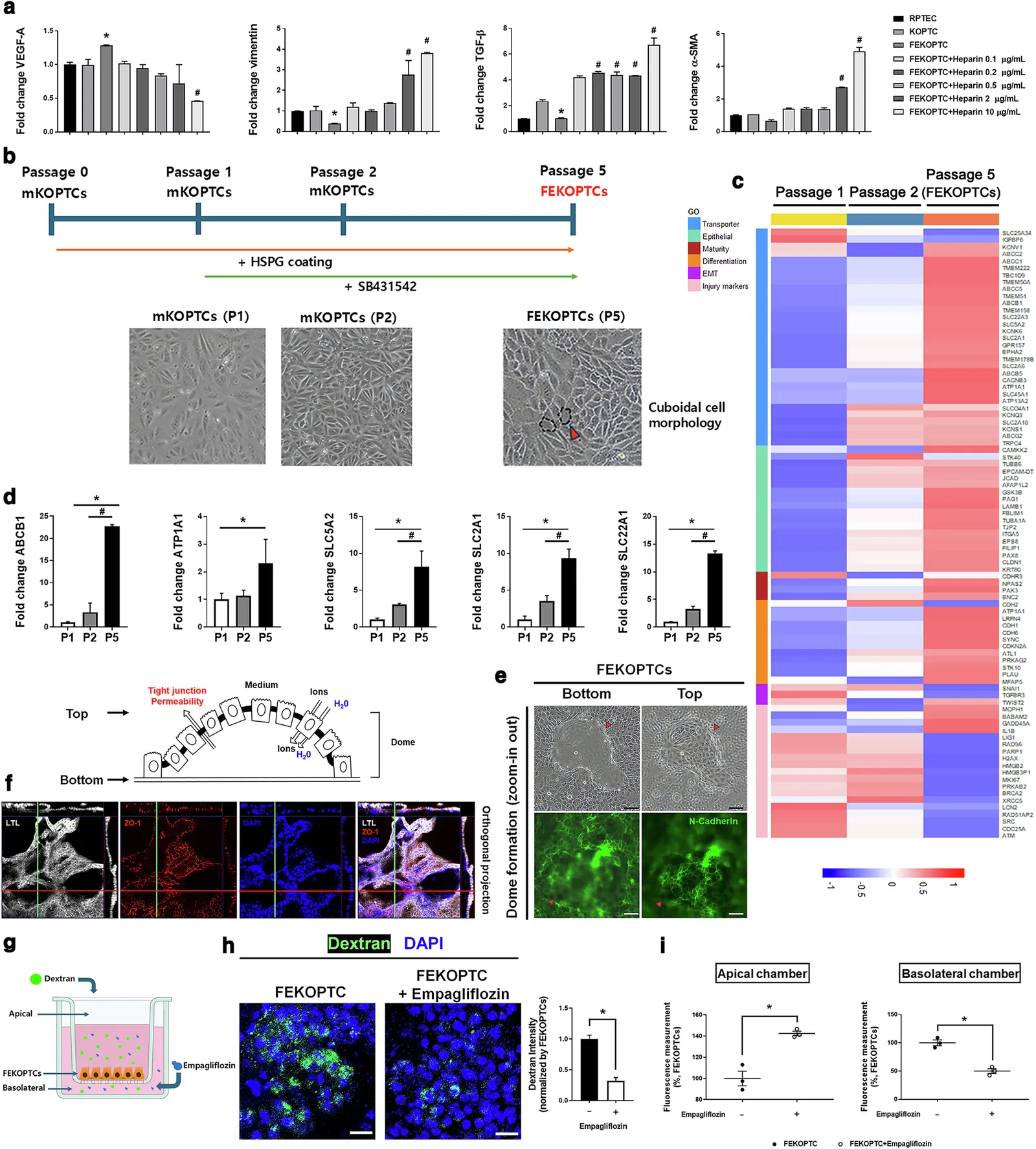
Generation of FEKOPTCs from mKOPTCs. **a** qRT-PCR analysis of VEGF-A and EMT markers in mKOPTCs and FEKPOTCs in the absence or presence of heparin. **b** Schematic overview of the protocol to generate FEKOPTCs from mKOPTCs through the passage. Brightfield images showing mKOPTCs passages 1, 2, and FEKOPTCs (mKOPTC passage 5). **c** Heatmap comparing the expression of transporter, epithelial, maturity, differentiation, EMT, and injury markers from passages 1 and 2 and FEKOPTCs (mKOPTC passage 5). **d** qRT-PCR analysis of ABCB1 (drug transporter gene), ATP1A1 (solute transporter gene), SLC5A2 and SLC2A1 (glucose transporter genes), and SLC22A1 (organic cation transporter 1 gene) among passages 1 and 2 and FEKOPTCs (mKOPTC passage 5). (four independent experiments, *n* ≥ 3 well per condition). Values are mean ± SEM. *, versus passage 1; #, versus passage 2 by the post hoc Mann–Whitney *U*-test. **e** When grown to confluence, FEKOPTCs exhibited a cobblestone shape and a multicellular dome formation (two top and bottom layers). **f** Representative confocal images showing 3D tubular structure in a Transwell system^TM^. The FEKOPTCs form a single-layered tubule encircling a lumen. The red and green line indicates an area of the *yz*-orthogonal projection. Immunofluorescence staining shows luminal localization of LTL (proximal tubule cell marker) and ZO-1 (tight junction protein). **g** Graphic summary of the Transwell system for an in vitro dextran uptake assay of FEKOPTCs. The 10-kDa FITC-dextran was treated in an apical chamber depicting a urinary space, and empagliflozin was treated in a basolateral chamber depicting a blood space. **h** Representative confocal images and quantification of the pixel intensity after incubation with dextran (10 kDa)-FITC in the presence or absence of empagliflozin. **i** Quantification of flux by the measurement of fluorescence in the apical chamber and the basolateral chamber after incubation with dextran (10 kDa)-FITC in the presence or absence of empagliflozin. Values are mean ± SEM. *, versus absence of empagliflozin by the post hoc Mann–Whitney *U*-test.

On the basis of Fig. 1a,b, we investigated whether pretreatment with HSPG affects the interaction of VEGF signaling, which is crucial for regulating the epithelial–mesenchymal transition (EMT) pathway^26^. As shown in Fig. 3a, VEGF expression significantly increased in FEKOPTC cells that were precoated with HSPG. By contrast, VEGF expression decreased significantly in a dose-dependent manner with heparin treatment (Fig. 3a). Additionally, EMT markers such as Vimentin, TGF-β, and α-SMA increased significantly following heparin treatment. These findings indicate that HSPG improves the maturity of mKOPTCs. The addition of SB431542, a selective TGF-β receptor inhibitor, was required for maintaining the cells in a differentiated and polarized phenotype after passaging for 1 day. In the absence of SB431542 treatment, cells rapidly altered their morphology from cobblestone-shaped cells to a fibroblast-like morphology within 1 day in culture (Supplementary Fig. 4).

Finally, we optimized culture conditions, as shown in Fig. 3b, by which LTL-positive cells had a cuboidal phenotype that closely mimicked the phenotype of PTCs and grew at a rapid rate (Fig. 3b). We called the cells, cultured using the protocol shown in Fig. 3b, FEKOPTCs.

Next, we examined the gene expression of the functional transporter of PTCs according to passage time. RT-PCR analysis revealed that the gene expressions of drug transporter genes (ABCB1), tubular epithelial solute transporters (ATP1A1), glucose transporters (SLC5A2 and SLC2A1), and organic cation transporter 1 (SLC22A1) were upregulated in passage 5 KOPTCs (FEKOPTCs) compared with those in passage 1 and passage 2 mKOPTCs (Fig. 3c). To determine the gene expression patterns according to passage time, we performed bulk RNA-seq at early passage (passage 1 and 2) and late passage (passage 5) (Fig. 3d). A heatmap of representative gene expression patterns suggested that in the expression of transporters and epithelial markers, maturity and differentiation were increased while EMT and injury markers were reduced in the passage 5 mKOPTCs (FEKOPTCs) compared with those in passage 1 and passage 2 mKOPTCs (Fig. 3d). Taken together, these transcriptional results imply that mKOPTCs can be serially passaged without loss of identity and become mature according to passage time. In particular, the functional transporters and maturity were substantially increased in FEKOPTCs.

Next, we assessed the morphology of FEKOPTCs. The dome formation reflects the functional plasma membrane polarization, the formation of tight junctions, and the transepithelial solute transport of PTCs^27^. FEKOPTCs sheets exhibited typical epithelial dome-like structures. These confluent monolayers of the bottom formed in 3–4 days, and multicellular domes of the top layer remained viable for up to 7–8 days as determined by immunofluorescent staining of N-cadherin, a marker for PTCs (Fig. 3e).

A 3D environment facilitates morphological changes and organotypic functions^28^. We performed the cultivation of FEKOPTCs in a 3D extracellular matrix-like environment by seeding them onto a layer of HSPG coating on a Transwell membrane to form a branched, interconnected structure (Fig. 3f). Interestingly, tubular structures were observed, the lumen of which was surrounded by a single layer of FEKOPTCs stained with LTL and ZO-1 (Fig. 3f). Orthogonal projection analyses revealed that the FEKOPTCs encircling lumens were highly polarized (Fig. 3f). FEKOPTCs could be maintained in these 3D culture conditions for up to 15 days without major morphological changes or overgrowth by proliferating cells. These data suggest that 3D culture of FEKOPTCs may recapitulate the tubular structures, similar to in vivo.

One of the major functions of human PTCs is the ability to reabsorb glucose by SGLT2 located in the apical membrane of PTCs, which is essential for the glucose metabolism. SGLT2 reabsorbs glucose from the urinary space of the tubular lumen and secretes it into the blood space of the capillary space. We showed the histological expression of SGLT2 in FEKOPTCs, which is similar to PTCs in vivo (Fig. 2e). Thus, we investigated whether FEKOPTCs exhibit physiologically relevant functions. We designed the Transwell system for an in vitro dextran uptake assay (Fig. 3g). The apical chamber of the Transwell mimics a urinary space of the lumen of the renal tubule in vivo, and the basolateral chamber mimics a blood space of the peritubular capillary of the kidney in vivo (Fig. 3g). The reabsorption function of FEKOPTCs was assessed by a 10 kDa dextran uptake for 24 h after treatment of 10 kDa dextran in the apical chamber (a urinary space). It resulted in an increased uptake of dextran in FEKOPTCs, which was inhibited when empagliflozin, an SGLT2 inhibitor, was treated in the basolateral chamber (a blood space) (Fig. 3h). Interestingly, empagliflozin treatment increased the measurement of dextran in the apical chamber (a urinary space) and decreased in the basolateral chamber (a blood space) (Fig. 3i). This finding indicates that empagliflozin inhibits SGLT2 and its function of glucose resorption in FEKOPTCs, which is physiologically relevant in the human kidney.

### FEKOPTCs have improved expression of transporters, MRP2/4-mediated excretion, and epithelial barrier function compared with RPTECs

**Fig. 4:**
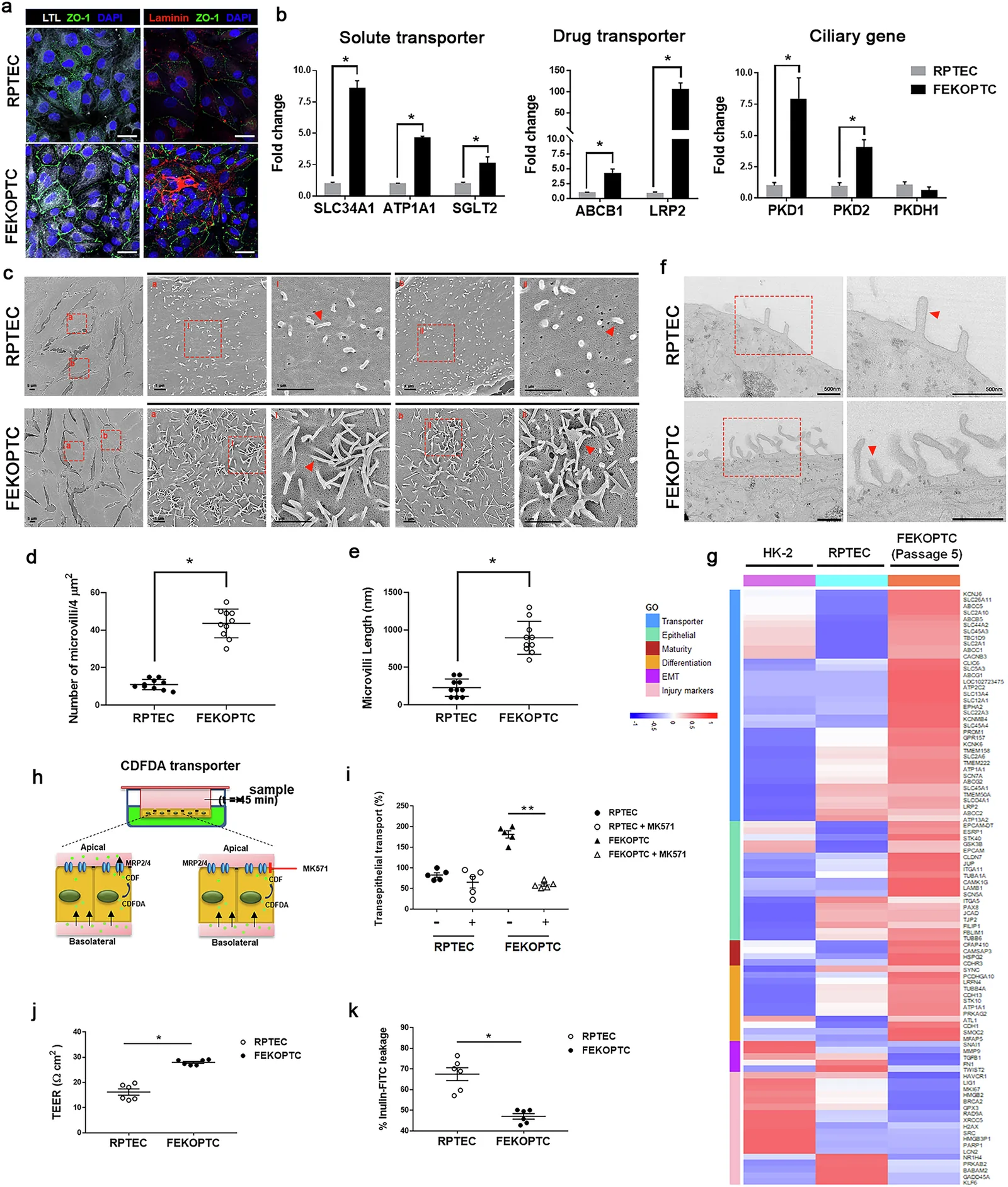
Comparison of morphology, gene expression, and functions between FEKOPTCs and RPTECs. **a** Representative confocal image from the apical side to the basal side shows the expression of an apical marker (ZO-1), laminin clusters, and a proximal tubule marker (LTL). Scale bar, 30 µm. **b** qRT-PCR analysis of tubular epithelial solute transporters (SLC34A1, ATP1A1, and SGLT2), drug transporters (ABCB1 and LRP2), and ciliary genes (PKD1, PKD2, and PKDH1) (*n* = 3). **c** Representative SEM images showing the microvilli on the apical surface between FEKOPTC and RPTECs. Scale bars, 5 µm and 1 µm. **d**,**e** Quantification of the number of microvilli and the length in SEM images (*n* = 10, 4 µm^2^). Values are mean ± SEM. *, versus RPTECs by the post hoc Mann–Whitney *U*-test. **f** Representative TEM images of RPTECs and FEKOPTCs. Scale bar, 500 nm. Red arrowheads indicated microvilli structures. **g** Heatmap comparing the expression of transporter, epithelial, maturity, differentiation, EMT, and injury markers among HK-2, RPTEC, and FEKOPTC. **h** Graphic summary of the transepithelial transport analysis and MRP2/4-mediated apical efflux using a Transwell system. **i** Comparison of the function of transepithelial transport and MRP2/4-mediated efflux between FEKOPTCs and RPTECs. **j** Comparison of TEER between FEKOPTCs and RPTECs. **k** Comparison of inulin-FITC leakage between FEKOPTCs and RPTECs (*n* = 3, four technical replicates each, analyzed together). Values are mean ± SEM. *, versus RPTECs by the post hoc Mann–Whitney *U*-test.

To investigate the potential of FEKOPTCs as an in vitro model of human PTCs, FEKOPTCs were compared with RPTECs, which are human primary PTCs used as an in vitro model of human PTCs. Confocal microscopy showed that the brush border marker LTL is expressed more strongly in FEKOPTCs than in RPTECs (Fig. 4a). FEKOPTCs secrete their own ECM laminin, and the tight junction protein, ZO-1, is more strongly expressed in FEKOPTCs than in RPTECs (Fig. 4a).

RT-PCR analysis showed that the gene expression of tubular epithelial solute transporters (SLC34A1, ABCB1, and ATP1A1), glucose and albumin transporters (SGLT2 and LRP2), and ciliary genes (PKD1 and PKD2) were upregulated in FEKOPTCs compared with those of RPTECs (Fig. 4b).

Further structural and phenotypic characterization of the tubular structures was achieved by electron microscopy and confocal imaging. We further analyzed SEM analysis of the comparison between RPTECs and FEKOPTCs, which confirmed a monolayer formation and showed brush border structures, including microvilli at the apical surface, indicating cellular polarity (Fig. 4c). Also, we analyzed this structure by TEM to determine the cellular ultrastructure, comparing the differences between RPTECs and FEKOPTCs (Fig. 4f).

In Fig. 4d,e, SEM analysis revealed that the number and length of microvilli were greater in FEKOPTCs compared with RPTECs. These findings are consistent with the TEM image (Fig. 4f) and indicate that polarization was more effectively established in FEKOPTCs than in RPTECs.

Confocal microscopy of a dome structure is shown in Supplementary Fig. 5, a *y*–*z* projection revealed that nuclei are arranged in a circular manner, conforming to the in vivo-like morphotype. (Supplementary Fig. 5) Renal proximal tubule epithelium expressed transporters on both apical and basolateral membranes, which collectively mediate the elimination of drugs. Using *z*-stack orthogonal projection analyses, the polarization of the 3D dome formation was stained to investigate the apical membrane localization of glucose transporter marker, sodium glucose co-transporter 2 (SGLT2), and investigate the basolateral membrane localization of organic anion transporters 1 and 3 (OAT1 and OAT3). On the Transwell system, confocal microscopy showed that OAT1 and OAT3 on the basolateral membrane are expressed more strongly in FEKOPTCs than in RPTECs (Supplementary Fig. 5a,b).

To compare the gene expression patterns among FEKOPTCs, RPTECs, and HK-2 cells, we performed bulk RNA-seq (Fig. 4g). A heatmap of representative gene expression patterns suggested that in the expression of transporters of PTCs and epithelial markers, maturity and differentiation were increased more, and EMT and injury markers were reduced more in FEKOPTC compared with those of RPTECs and HK-2 cells (Fig. 4g).

5(6)-Carboxy-2’,7’-dichlorofluorescein diacetate (CDFDA) diffuses into PTCs and is hydrolyzed to CDF, a substrate of the multidrug resistance protein 2 and 4 (MRP2/4) efflux pumps that remove xenobiotics in the human kidney^29^. We assessed the capability of selective excretion by exposure to CDFDA in the basolateral aspect of the cells (Fig. 4h,i). Using a Transwell system, transepithelial transport and MRP2/4-mediated apical efflux were analyzed (Fig. 4h). After 45 min of exposure to CDFDA, CDF was detected apically in both FEKOPTCs and RPTECs but was detected at higher levels in FEKOPTCs (Fig. 4i). MK571, an MRP2/4 inhibitor, inhibited the luminal efflux of CDF. Treatment with MK571 significantly decreased MRP2/4-mediated excretion in FEKOPTCs compared with RPTECs (Fig. 4i). These findings indicate the higher capability of MRP2/4-mediated excretion of FEKOPTCs.

In the assessment of the epithelial barrier function, FEKOPTCs had higher TEER and significantly lower leakage of 2–5-kDa inulin-FITC than RPTECs, which indicates that FEKOPTCs had better epithelial barrier function and leak tightness and were suitable for the study of tubular paracellular leakage in vitro (Fig. 4j,k).

Taken together, these findings suggest that FEKOPTCs might be a useful in vitro model of human PTCs.

### Similarity of FEKOPTCs and PTCs of native human adult kidney

**Fig. 5:**
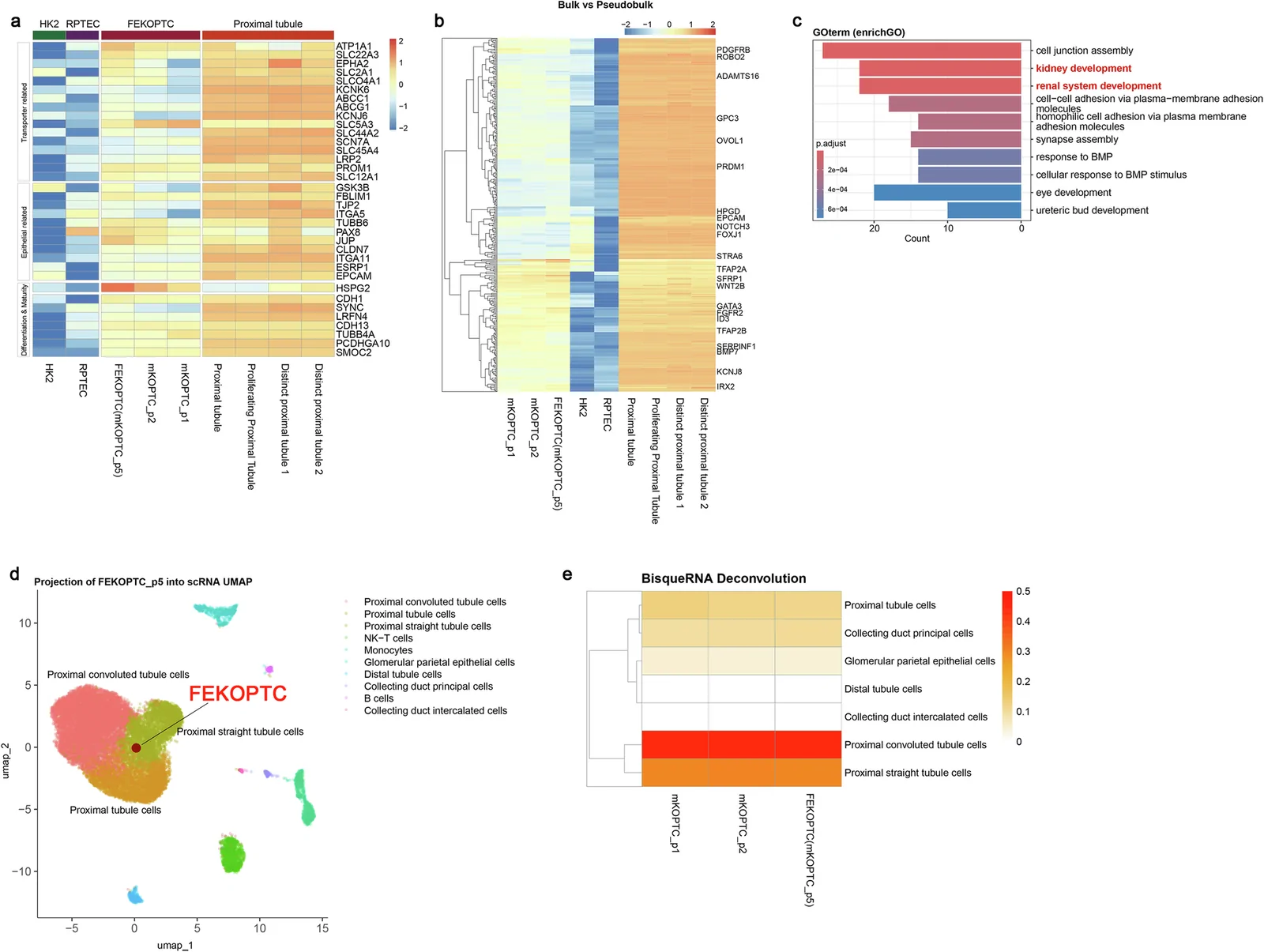
Transcriptomic characterization of FEKOPTCs reveals high similarity to PTCs of native human adult kidney. **a** Heatmap showing gene expression profiles between bulk RNA-seq of FEKOPTCs, RPTEC, and HK2, and pseudo-bulk transcriptomes derived from proximal tubule-related clusters in the mature human kidney single-cell reference dataset. **b** Heatmap of 300 genes that are highly expressed in both FEKOPTCs and proximal tubule pseudo-bulk references, but underexpressed in RPTEC and HK2. **c** Gene Ontology (GO) enrichment analysis of the 300 selected genes, showing significant association with kidney-specific developmental processes. **d** UMAP projection of FEKOPTC bulk RNA-seq data (red dot) onto the single-cell transcriptomic landscape of human kidney (GSE131685). **e** Estimated cellular composition of FEKOPTC as determined by BisqueRNA deconvolution using the GSE131685 reference.

To assess the similarity between FEKOPTCs and PTCs of the adult human kidney, we compared bulk RNA-seq profiles from FEKOPTCs with pseudobulk transcriptomes generated from PT-related clusters (Proximal tubule, Proliferating proximal tubule, Distinct proximal tubule 1, and Distinct proximal tubule 2) in the native human adult kidney single-cell reference dataset (www.kidneycellatlas.org). Heatmap analysis showed that FEKOPTCs exhibited greater transcriptional similarity to in vivo PT clusters than other immortalized PT-like cell lines, such as RPTEC and HK-2, suggesting improved transcriptional fidelity to the native adult PT identity (Fig. 5a).

We further analyzed 300 genes that were co-expressed in both FEKOPTC and single-cell-derived PT pseudo-bulk reference, but distinctly underexpressed in RPTEC and HK2 (Fig. 5b). Functional enrichment analysis using enrichGO revealed that these genes were significantly associated with kidney-specific biological processes, including kidney development and renal system development (Fig. 5c). These findings suggest that FEKOPTCs more closely recapitulate the native PT transcriptional program and may provide a more physiologically relevant model for studying human kidney biology.

To further validate this similarity, we projected FEKOPTC bulk RNA-seq data onto UMAP embedding of a publicly available human kidney single-cell RNA-seq dataset (GSE131685)^30^. The FEKOPTC localized centrally within the PT cluster region, showing their transcriptional resemblance to in vivo PT cells (Fig. 5d).

In addition, deconvolution analysis using BisqueRNA further supported this observation. When FEKOPTC expression data were deconvolved using the single-cell reference, the inferred cellular composition was predominantly composed of proximal convoluted tubule cells (~44%), proximal straight tubule cells (~30%), and proximal tubule cells (~13%) (Fig. 5e). These results confirm that FEKOPTCs retain key transcriptional programs characteristic of PT cells and further support their utility as an in vitro model for studying human kidney proximal tubule biology.

### Nephrotoxicity test using FEKOPTCs-on-chip

**Fig. 6:**
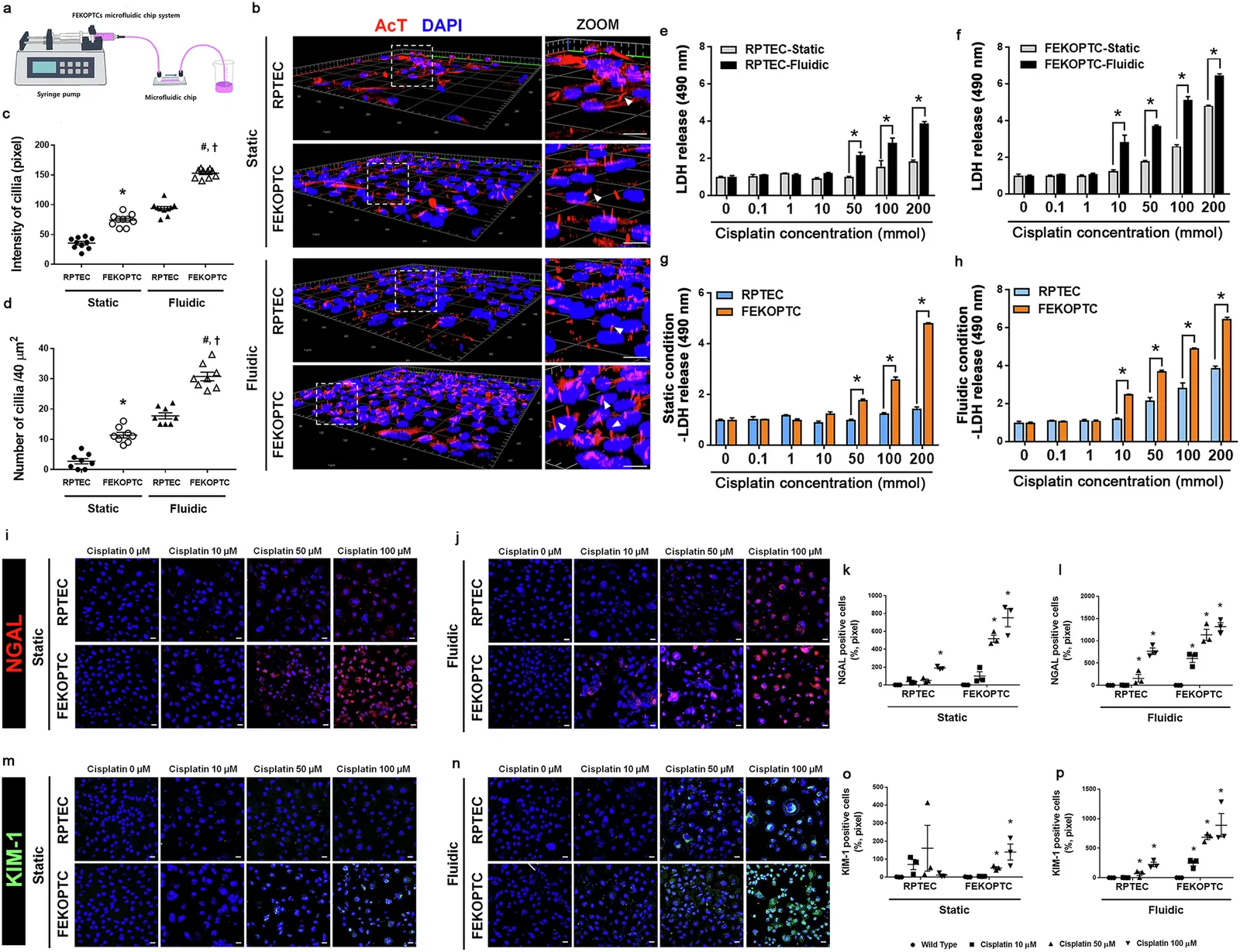
Nephrotoxicity testing using FEKOPTCs-on-chip. **a** Schematic drawing of the FEKOPTC-on-chip with microfluidic chip system. A diagram showing the FEKOPTCs or RPTECs cultured on the microfluidic chip. **b** Immunofluorescent staining of FEKOPTCs and RPTECs cultured on microfluidic chips and static conditions for 10 days, to show AcT (acetylated tubulin) as a polarization marker. **c**,**d** Quantification of the pixel intensity and number of cilia stained with AcT. White arrows indicated cilia of cells. Scale bar, 20 µm. **e**,**f** Comparison of the relative release of normalized LDH after treatment with cisplatin for 48 h between static condition and microfluidic condition in FEKOPTCs-on chip and RPTECs-on chip, respectively (*n* = 3, three technical replicates each, analyzed together). **g**,**h** Comparison of the relative release of normalized LDH after treatment with cisplatin for 48 h between FEKOP TCs-on chip and RPTECs-on chip in the static condition and fluidic condition, respectively (*n* = 3, three technical replicates each, analyzed together). Values are mean ± SEM. *, versus RPTECs by the post hoc Mann–Whitney *U*-test. **i–l** Representative images of NGAL expression in FEKOPTCs on chip versus RPTECs on chip after treatment with cisplatin for 48 h in static and microfluidic chip conditions. Quantification of the percentage of NGAL-positive cells (*n* = 3, three technical replicates each, % pixel). **m–p** Representative images of KIM-1 expression in FEKOPTCs on chip versus RPTECs on chip after treatment with cisplatin for 48 h in static and microfluidic chip conditions. Quantification of the percentage of KIM-1-positive cells (*n* = 3, three technical replicates each, % pixel).

Next, we create FEKOPTC-on-chip models for the nephrotoxicity test. In brief, FEKOPTCs were seeded onto an integrated multiplexed platform composed of a microfluidic chip by the syringe pump and cultured for more than 1 week (Fig. 6a). To validate the efficacy of the nephrotoxicity test using FEKOPTCs-on-chip, we compared it with RPTECs-on-chip, which were manufactured in the same manner as FEKOPTCs-on-chip, except that RPTECs were used instead of FEKOPTCs.

After 10 days of dynamic cultivation, the polarization status of the cells on the chips was assessed. Acetyl-α-tubulin (AcT) is found predominantly in the primary cilium, a mechano-sensing organelle present in PTCS, which is a marker of the polarization of PTCs toward the tubular lumen because it is located in the apical part of PTCs. Immunofluorescence analysis showed that the number and length of AcT-stained cilia were higher in FEKOPTCs-on-chip compared with those of RPTECs-on-chip, both in a static condition, and were further enhanced in a fluidic condition (Fig. 6b–d). These findings suggest that polarization was most effectively achieved in FEKOPTCs-on-chip under the fluidic condition.

Cisplatin is a metal complex with anticancer activity, and it is a broad-spectrum anticancer drug used in clinics. It can be absorbed by organic cation transporters (OCTs) and the multidrug and toxin extrusion (MATE) family of transporters in the PTCs and leads to cumulative nephrotoxicity with the production of reactive oxygen species^31^. To determine the ability of FEKOPTCs for nephrotoxicity tests, we performed a nephrotoxicity test of cisplatin using these systems.

Cisplatin was introduced through the channel on FEKOPTCs-on-chip and RPTECs-on-chip and allowed to perfuse for 48 h on the chip device in both static and microfluidic conditions. Luminal tubing media samples were collected, and an LDH assay was performed. As the concentration of cisplatin increased, the normalized LDH release was significantly higher in the fluidic condition compared with the static condition on both FEKOPTCs-on-chip and RPTECs-on-chip (Fig. 6e,f). A difference in normalized LDH response between the static condition and fluidic condition was observed upon exposure to 10 µM cisplatin in FEKOPTCs-on-chip, whereas it was observed upon exposure to 50 µM cisplatin in RPTECs-on-chip (Fig. 6g,h). These findings suggest that both chips are more sensitive to the detection of nephrotoxicity in the fluidic condition compared with the static condition, which is enhanced in FEKOPTCs-on-chip. Figure 6f,h shows that FEKOPTCs-on-chip exhibited a significantly higher normalized LDH response compared with RPTECs-on-chip, which was observed upon exposure to a lower concentration of cisplatin in the fluidic condition compared with the static condition. These observations suggest that FEKOPTCs-on-chip in a fluidic condition is the most sensitive predictor of toxicity in PTCs following cisplatin treatment.

Neutrophil gelatinase-associated lipocalin (NGAL) and kidney injury molecule-1 (KIM-1) are expressed in PTCs after AKI and are markers of damage to the PTCs^32^. They are biomarkers for early diagnosis of acute kidney injury (AKI), including cisplatin-induced AKI^32,33^. Confocal microscopy analysis revealed that the expression of NGAL began at a cisplatin concentration of 50 µM and increased with concentration in FEKOPTCs-on-chip, while it began at a cisplatin concentration of 100 µM in RPTECs-on-chip in the static condition (Fig. 6i,k). In the fluidic condition, the expression of NGAL started from exposure to 10 µM cisplatin in FEKOPTCs-on-chip, whereas it started from exposure to 50 µM cisplatin in RPTECs-on-chip in the static condition (Fig. 6j,l). Similar data were observed in KIM-1 expression (Fig. 6m–p). These findings are consistent with the results of the normalized LDH assay.

The IC50 is the specific concentration of a substance required to kill or inhibit 50% of the cells, which is a common and useful metric for assessing drug cytotoxicity. IC50 can vary significantly across cell lines. We compared the IC50 of cisplatin between FEKOPTCs and RPTECs. Our data showed that FEKOPTC has an IC50 of 38.85 µM, whereas RPTEC has an IC50 of 71.49 µM (Supplementary Fig. 6), which indicates that FEKOPTC is more sensitive to cisplatin-induced cytotoxicity compared with RPTEC. These findings are consistent with the results of the normalized LDH assay and NGAL and KIM-1 assay.

Taken together, these findings imply that FEKOPTCs-on-chip in the fluidic condition is a sensitive predictor to detect the damage of PTCs and might be suitable for testing the nephrotoxicity of drugs.

### FEKOPTCs modeled Fabry disease by using CRISPR–Cas9 genetic mutation of GLA

Fabry disease is a rare X-linked inherited disorder that causes defects in the glycosphingolipid metabolic pathway that result from deficient or absent activity of the lysosomal enzyme, GLA, which leads to the accumulation of globotriaosylceramide (Gb3) and related neutral glycosphingolipids within lysosomes^34,35^. The accumulation of Gb3 results in impairment of cellular morphology and function^34,36^. Fabry nephropathy results from the accumulation of Gb3 in kidney cells that include tubular epithelial cells, podocytes, mesangial cells, and vascular endothelial cells^37,38^. Renal involvement of Fabry disease is the primary cause of death in patients with untreated Fabry disease^39,40^. To develop new therapies to cure the disease, human experimental models of Fabry nephropathy are needed.

**Fig. 7:**
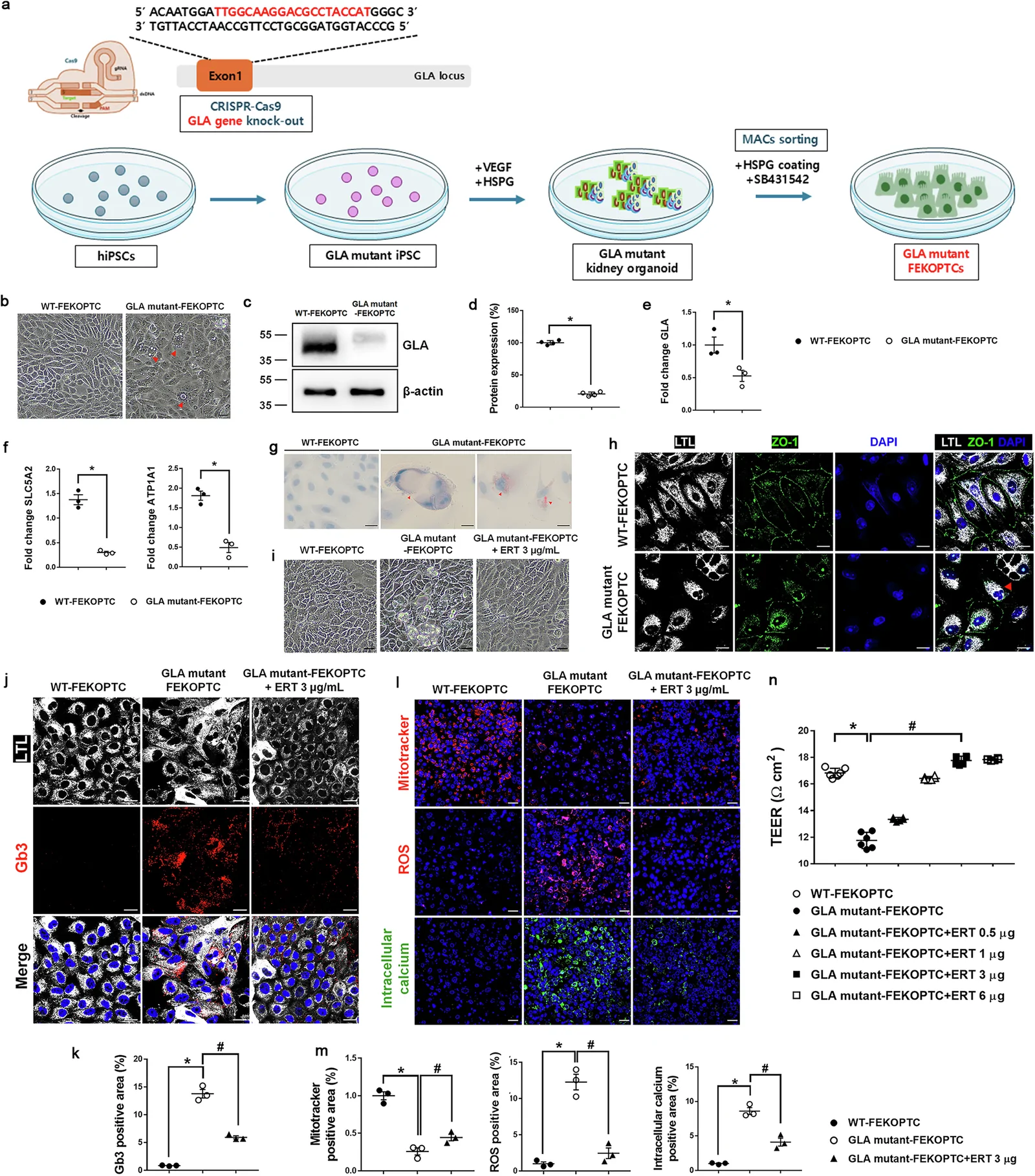
Modeling of Fabry nephropathy using FEKOPTCs through CRISPR–Cas9 genetic editing. **a** Schematic overview of the process used to establish GLA-mutant FEKOPTCs. **b** Representative brightfield images of 2D cell monolayers of wild-type (WT) FEKOPTCs and GLA-mutant FEKOPTCs. The lipid droplets are shown in GLA-mutant KOPTCs (red arrowhead). Scale bar, 50 µm. **c**,**d** Representative immunoblot of the expression of GLA in WT-KOPTCs and GLA-mutant FEKOPTCs. **e**,**f** qRT-PCR analysis of GLA-mutant FEKOPTCs. **g** Representative Oil Red O staining images showing lipid droplet formation (red arrowhead) in GLA-mutant FEKOPTCs. **h** Confocal images of WT-FEKOPTCs and GLA-mutant FEKOPTCs showing LTL, ZO-1, and intracellular lipid droplets (red arrowhead). **i** Representative brightfield image of changes in the morphology of GLA-mutant FEKOPTCs after ERT. Scale bar, 50 µm. **j** Confocal images showing immunofluorescence staining of Gb3 in WT-FEKOPTCs, GLA-mutant FEKOPTCs, and GLA-mutant FEKOPTCs treated with ERT. Scale bar, 20 µm. **k** Quantification of the percentage of Gb3-positive area. Values are mean ± SEM. *, WT-FEKOPTCs versus GLA-mutant FEKOPTCs; #, GLA-mutant KOPTCs versus GLA-mutant KOPTCs treated with ERT by the post hoc Mann–Whitney *U*-test. **l** Representative image of immunofluorescence staining to show MitoTracker, MitoSox red, and Intracellular calcium, in WT-FEKOPTCs, GLA-mutant FEKOPTCs, and GLA-mutant FEKOPTCs treated with ERT. Scale bar, 50 µm. **m** Quantification of intracellular calcium by analyzing the fluorescence intensity of Fluo-8, MitoSox red, and MitoTracker. Values are mean ± SEM. *, WT-KOPTCs versus GLA-mutant FEKOPTCs; #, GLA-mutant FEKOPTCs versus GLA-mutant FEKOPTCs treated with ERT by the post hoc Mann–Whitney *U*-test. **n** Measurement of TEER in GLA-mutant FEKOPTCs treated with ERT. Values are mean ± SEM. *, WT-FEKOPTCs versus GLA-mutant FEKOPTCs; #, GLA-mutant FEKOPTCs versus GLA-mutant FEKOPTCs treated with ERT by the post hoc Mann–Whitney *U*-test.

To determine the ability of FEKOPTCs to model kidney disease, we examined whether FEKOPTCs could model Fabry disease. Figure 7a shows a schematic drawing of the process used to generate GLA-mutant FEKOPTCs. In brief, we generated it in human iPSCs using CRISPR/Cas9-mediated gene editing and differentiated kidney organoids (GLA-mutant kidney organoids) by the protocol shown in Fig. 1c (Fig. 1c and Supplementary Fig. 7). Then, PTCs were isolated from GLA-mutant kidney organoids and cultivated by the protocol shown in Fig. 3b, which finally resulted in the generation of GLA-mutant FEKOPTCs.

Brightfield microscopy revealed that GLA-mutant FEKOPTCs had deformed structures with irregular shapes, whereas wild-type (WT)-FEKOPTCs had well-differentiated cuboidal shapes (Fig. 7b). Immunoblotting confirmed the existence of frameshift mutations at the target site and the absence of the corresponding full-length proteins (Fig. 7c,d). We also confirmed significantly decreased mRNA expression of GLA in GLA-mutant FEKOPTCs compared with those of WT-FEKOPTCs (Fig. 7e). These findings indicated the successful genetic mutation of GLA in GLA-mutant FEKOPTCs.

Next, we examined whether GLA-mutant FEKOPTCs might produce phenotypes relevant to human Fabry nephropathy. The gene expression of PTCs was downregulated in GLA-mutant FEKOPTCs compared with WT-FEKOPTCs (Fig. 7f). Oil Red O staining revealed abundant lipid droplet-like deposits in the cytoplasm observed in GLA-mutant FEKOPTCs (Fig. 7g). ZO-1 expression as an indicator of tight-junction integrity and maintenance of epithelial organization was significantly reduced in GLA-mutant FEKOPTCs compared with that of WT-KOPTCs (Fig. 7h). Confocal microscopy revealed the extensive accumulation of Gb3, a main pathomechanism in human Fabry disease, in GLA-mutant FEKOPTCs (Fig. 7j). These data indicated that GLA-mutant FEKOPTCs have the ability to phenocopy human Fabry nephropathy.

### GLA-mutant FEKOPTCs recapitulate the therapeutic efficacy of ERT for Fabry nephropathy

ERT with enzyme-recombinant human alpha-galactosidase (rhα-GLA) is a primary treatment option for patients with Fabry disease^41^. ERT improves Fabry-related symptoms and slows renal damage when started at an earlier stage^41^. Therefore, we examined whether GLA-mutant FEKOPTCs could recapitulate the therapeutic efficacy of ERT with rhα-GLA. ERT restored the deformed cellular structure of GLA-mutant FEKOPTCs to a more organized pattern (Fig. 7i). ERT also decreased the accumulation of Gb3 of GLA-mutant FEKOPTCs (Fig. 7j,k).

The accumulation of Gb3 is induced by increased oxidative stress and reactive oxygen species (ROS) in Fabry nephropathy, which may be attenuated by ERT^42^. Thus, we examined the effect of ERT on oxidative stress in GLA-mutant FEKOPTCs (Fig. 7l,m). The intensity of Mitotracker decreased in GLA-mutant FEKOPTCs and was recovered after ERT (Fig. 7l,m). The superoxide in the mitochondria was examined using MitoSox Red staining. MitoSox fluorescence increased in GLA-mutant FEKOPTCs and remained but decreased after ERT (Fig. 7l,m). Oxidative stress and ROS result in an increase in calcium influx into the cytoplasm. We examined intracellular calcium by staining with the calcium-sensitive fluorescent dye fluo-8 (Fig. 7l,m). Fluo-8-positive cells increased in GLA-mutant FEKOPTCs. After ERT, intracellular calcium decreased (Fig. 7l,m). These findings suggest that GLA-mutant FEKOPTCs could recapitulate the effect of ERT on oxidative stress in Fabry nephropathy.

Additionally, we performed a TEER analysis to determine whether GLA-mutant FEKOPTCs recapitulate physiologically relevant features or are attenuated by ERT. WT-FEKOPTCs achieved a steady-state TEER of 10–20 Ω·cm^2^ when grown on 12-well Transwell filters (Fig. 7n). GLA-mutant FEKOPTCs had significantly decreased TEER compared with WT-FEKOPTCs, which were significantly increased in a dose-dependent manner by ERT treatment (Fig. 7n).

Taken together, these findings suggest that GLA-mutant FEKOPTCs’ ability to model Fabry disease might be a valuable tool for identifying the mechanisms and development of novel therapeutic alternatives for Fabry nephropathy.

### FEKOPTCs transplanted into the kidney of NOD-SCID mice were reassembled to form proximal tubule-like structures

We and others previously reported that the transplantation of kidney organoids under the kidney capsule enhanced vascularization and maturation more than kidney organoids grown in vitro^9,43–47^. Transplantation experiments may be important to determine regenerative potential in vivo. However, most transplantation experiments have been performed with a method using transplantation under the kidney capsule. This method has failed to establish a connection between the tubules in the transplanted kidney organoids and the tubules of the host mouse kidney, which may be a limitation of kidney organoids as a kidney regenerative cell therapy.

**Fig. 8:**
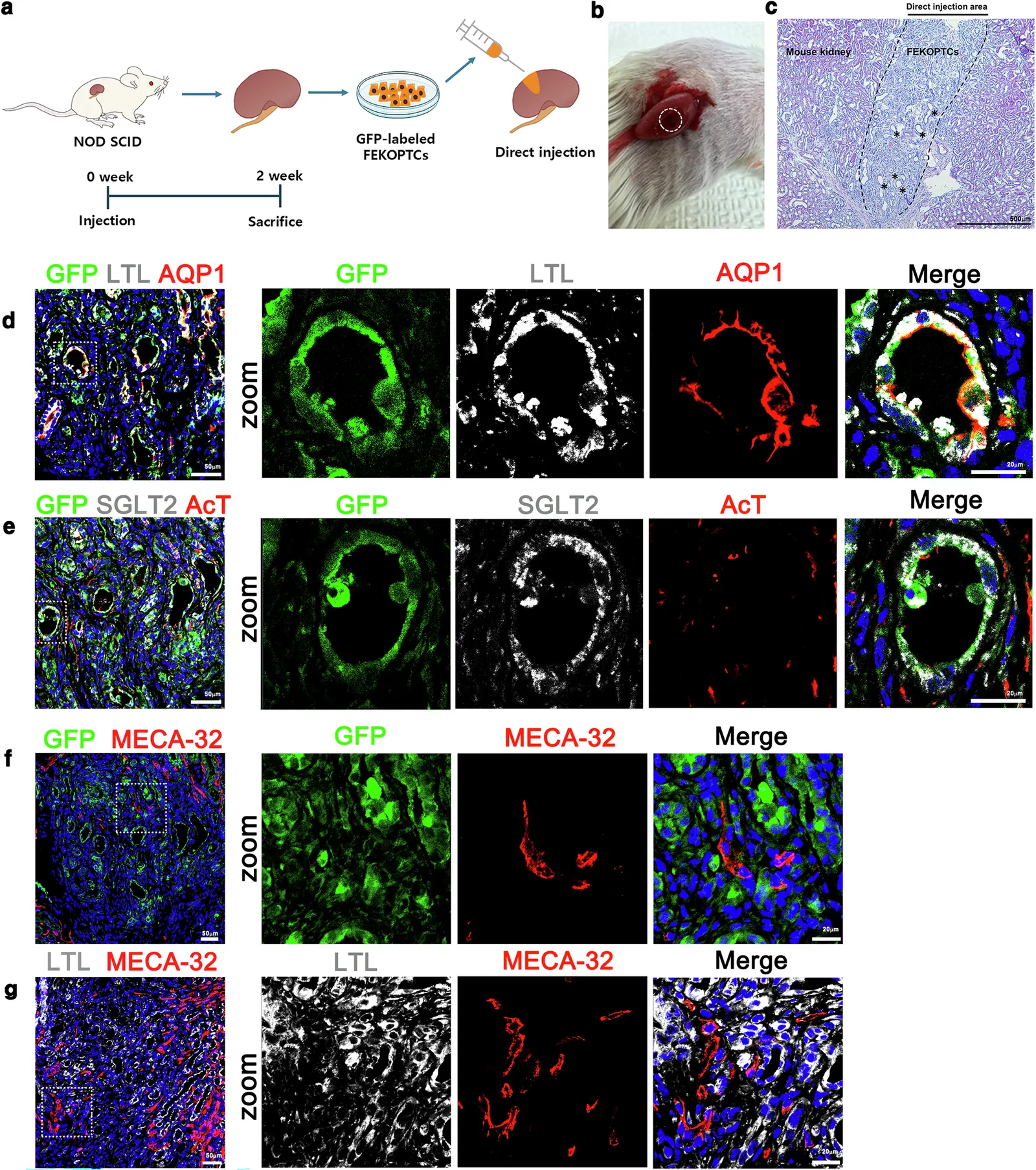
Transplantation of FEKOPTCs into the parenchyma of the NOD-SCID mouse kidney. **a** Schematic overview of the transplantation of GFP-labeled FEKOPTCs into the parenchyma of immunodeficient NOD-SCID mouse kidney. **b** Representative images of the host mouse kidney transplanted with FEKOPTCs. **c** Representative images of hematoxylin and eosin staining showing the transplanted KOPTCs into the parenchyma of the host mouse kidney. Scale bar, 500 µm. **d**,**e** Representative confocal images of GFP (GFP-labeled FEKOPTCs), LTL (proximal tubule marker), AQP1 (water channel), SGLT2 (glucose transporter), and AcT (Ciliary protein) of the graft in the host mouse kidney. Scale bars, 50 µm and 20 µm. **f**,**g** Representative confocal images of GFP (GFP-labeled FEKOPTCs), LTL (proximal tubule marker), and MECA-32 (endothelial cell) of the graft in the host mouse kidney. Scale bars, 50 µm and 20 µm.

In this study, to examine the regenerative potential of FEKOPTCs, we transplanted GFP-labeled FEKOPTCs into the kidney parenchyma rather than under the kidney capsule of immunodeficient NOD-SCID mice (Fig. 8a,b). A large number of tubule-like structures were observed in the kidney parenchyma of the host mice in H&E staining 2 weeks after transplantation (Fig. 8c). The tubule-like structures in the graft transplanted with FEKOPTCs expressed GFP, which suggests that the transplanted FEKOPTCs reassembled in the host kidney parenchyma to form the tubule-like structures (Fig. 8c). Interestingly, GFP-labeled cells of the tubule-like structures in the graft were expressed as the markers and transporters of PTCs including LTL, AQP1, and SGLT2 (Fig. 8d). Furthermore, they also expressed AcT toward the tubular lumen (Fig. 8e). These findings suggest that tubule-like structures in the graft consist of cells that have the characteristics of PTCs and are well polarized.

Vascularization of the graft is important for graft survival and function. MECA-32, a marker of host mouse endothelial cells, was abundantly observed in the graft transplanted with FEKOPTCs (Fig. 8f,g). This finding suggests that endothelial cells from the host mouse kidney had extensively invaded the graft and formed a vascular network.

Taken together, our data suggest the potential of FEKOPTCs as a regenerative cell therapy.

## Discussion

PTCs perform their functions by reabsorbing more than two-thirds of the sodium, water, amino acids, and essential nutrients (such as glucose, albumin, and bicarbonate) that are filtered from the glomeruli^48,49^. To perform those functions, PTCs express numerous solute transporters on their apical and basolateral membranes and have abundant mitochondria to support those energy-intensive transporters^50^. Because the kidneys are responsible for metabolizing and eliminating various drugs, this transportation system inevitably makes PTCs particularly susceptible to drug exposure^51^. For this reason, PTCs are the primary site of injury in AKI, especially drug-induced AKI.

Therefore, in vitro generation of PTCs that accurately reproduce the anatomy, function, and pathophysiology of PTCs in human kidneys would be valuable for predicting drug-induced AKI during drug development, modeling AKI in various disease conditions, and researching AKI mechanisms^52^.

Commercialized RPTECs and HK2 cells are widely used as cell sources for PTCs. The sensitivity and balanced accuracy of in vitro systems of PTCs for drug screening or nephrotoxicity testing are within the range of 70–90%, depending on the PTC type^53–56^. RPTECs, which are isolated primary human PTCs, have transepithelial transporters and express metabolic enzymes, and they are available for a broad range of biomarker assays. RPTECs are considered to be suitable for nephrotoxicity testing with high predictivity^57^. However, RPTECs rapidly dedifferentiate and senesce when they are cultured in isolation and lose the expression of key transporters and metabolic enzymes; furthermore, there is donor-to-donor variability and possible contamination with other kidney cell types during isolation from donors owing to the heterogeneity of kidneys^9,57,58^. HK2 cells, an immortalized human PTC line, also have limited clinical use because they have few transporters or proximal tubule characteristics and tend to lose their transepithelial transport by undergoing the EMT^59–61^.

In this study, we generated FEKOPTCs, functional PTCs derived from human iPSC kidney organoids. FEKOPTCs have improved epithelial barrier function, polarization state, and transporter function compared with RPTECs. Our data showed the potential of FEKOPTCs-on-chip as a drug toxicity test system by sensitively expressing NGAL and KIM-1 after exposure to nephrotoxic drugs. Considering that NGAL and KIM-1 are early biomarkers of AKI and are widely used in clinics to diagnose AKI, FEKOPTCs-on-chip may be an effective system to detect the early phase of AKI of nephrotoxic drugs and toxins.

Another interesting finding in this study is that FEKOPTCs can model kidney diseases. Kidney organoids are good tools for modeling kidney diseases. Using human kidney organoids, we previously modeled genetic kidney diseases such as focal segmental glomerulosclerosis, Fabry disease, and polycystic kidney disease^5,11,42^. Kidney organoids are a potentially powerful tool for high-throughput screening (HTS) for drug development^62^. However, HTS requires a large number of kidney organoids. Kidney organoids are difficult to cryopreserve and subculture. For these reasons, a large number of kidney organoids must be differentiated simultaneously each time HTS is performed. This may limit the establishment of an efficient HTS system using kidney organoids.

In this study, we have shown that FEKOPTCs can recapitulate the phenotype of Fabry kidney disease with CRISPR–Cas9 gene editing. FEKOPTCs have some advantages in kidney disease modeling over kidney organoids. Unlike kidney organoids, FEKOPTCs can be cryopreserved and serially passaged without loss of identity. Thus, FEKOPTCs that model kidney disease with CRISPR–Cas9 gene editing can thus be expanded with serial passaging and cryopreserved for HTS, making them available from the standard cell culture systems accessible to most researchers.

Another interesting point of this study is that FEKOPTCs could be good cell sources for regenerative medicine. We and others previously reported that kidney organoids transplanted beneath the kidney capsules of immunodeficient mice became vascularized from the host mouse endothelial cells and that the nephron-like structures in the grafts matured more than kidney organoids in vitro^9,11,43–47^. However, the transplanted kidney organoids remained immature. In fact, immaturity was a common feature of three separate classical differentiation protocols for kidney organoids^9,63^. Furthermore, the tubules in kidney organoids transplanted beneath the kidney capsules did not connect with the tubules in the neighboring host mouse kidney^9^. These findings weaken the regenerative potential of kidney organoids. In this study, we transplanted FEKOPTCs into the kidney parenchyma rather than beneath the kidney capsule. The transplanted FEKOPTCs reassembled in the kidney of the host mouse and constructed proximal tubular structures with intact lumens. The proximal tubules reconstructed by FEKOPTC transplantation expressed AQP1, which was not expressed in the kidney organoids transplanted beneath the kidney capsule^9^. These findings indicated the regenerative potential of FEKOPTCs.

Taken together, our data show that FEKOPTCs are functionally mature and can be applied to nephrotoxicity testing, disease modeling, and regenerative medicine.

## Supplementary information

Supplementary Information

## Data Availability

The data that support the findings of this study are available from the corresponding author upon reasonable request.
