# Supplementary material for: Generation of the functionally enhanced proximal tubular cells from human iPSC-derived kidney organoids
Source: Exp Mol Med. 2026 Sep 3;58(9):2972–89. doi: 10.1038/s12276-026-01821-0 (PMC13633281; doi:10.1038/s12276-026-01821-0)
Supplement: Supplementary file 1 [file 12276_2026_1821_MOESM1_ESM.pdf]

Supplementary Information

Generation of the functionally enhanced proximal tubular cells from human iPSC-derived kidney organoids

Sua Kim[1,2]* ,Sun Ah Nam[1]*, Jin Won Kim[1]* ,Jawoon Yi[4], Jin Ju Woo[1], Minji Lee[1], Jeongeun Lee[1], Tae-Hyung Kim[3], Hyunglae Kim[5], Hyung Wook Kim[5], Jiwhan Park[4], Yong Kyun Kim[1,2,5]

[1] Cell Death Disease Research Center, College of Medicine, The Catholic University of Korea, Seoul, Korea, [2] Rgenorgano Biotechnology, Seoul. Korea, [3] Department of Intelligent Precision Healthcare Convergence, Institute for Cross-disciplinary Studies (ICS), Sungkyunkwan University (SKKU), Suwon, Korea, [4] School of Life Sciences, Gwangju Institute of Science and Technology (GIST), Gwangju, Korea, [5] Department of Internal Medicine, The Catholic University of Korea, St. Vincent's Hospital, Seoul, Korea.

Supplementary Table 1. Primer sequences for qPCR

Gene	Forward (F) and reverse (R) primer sequences
**PKD1**	F 5´-AACAAGTCTTTGGCCATCAC-3´<br>R 5´-TACTCGTTCAGCACGGTGAC-3´
**PKD2**	F 5´-TCTTGCCAATTTCAGGCCTTT-3´<br>R 5´-GCACAACGATCACAACATCC-3´
**PKDH1**	F 5´-CCATTCTCTGCCAGGTTAGC-3´<br>R 5´-ACCCCTAATCAGCACAGTGG-3´
**SLC34A1**	F 5´-TCACGAAGCTCATCATCCAG-3´<br>R 5´-TTCCTCAGGGACTCATCACC-3´
**ATP1A1**	F 5´-CCAATTGTGTTGAAGGCACC-3´<br>R 5´-CCGTGATGATGTGGATAAAATGT-3´
**ABCB1**	F 5´-CCATGCTCAGACAGGATGTG-3´<br>R 5´-TTCCTGTCCCAAGATTTGCT-3´
**LRP2**	F 5´-TGTGATGCAGCCATCGAACT-3´<br>R 5´-TGCATTTGGGGAGGTCAGTC -3´
**SGLT2**	F 5´-GGGTTACGCCTTCCACGAG-3´<br>R 5´-AGATGTTTCCCACGGCTGG-3´
**PECAM1**	F 5´-TCATTACGGTCACAATGACGA-3´<br>R 5´-GAGTATCTGCTTTCCACGGC-3´
**PODXL**	F 5´-TCATTACGGTCACAATGACGA-3´<br>R 5´-GAGTATCTGCTTTCCACGGC-3´
**NPHS1**	F 5´-GACCCAGCTTCCCATCACTA-3´<br>R 5´-GCATTGGAGAGGAGCAGAAG-3´
**NPHS2**	F 5´-CTGTGAGTGGCTTCTTGTCCTC-3´<br>R 5´-CCTTTGGCTCTTCCAGGAAGCA-3´
**SYNPO**	F 5´-GCCGCAAATCCATGTTTACT-3´<br>R 5´-CTCATCCGCTGTCTGTACCA-3
**GGT1**	F 5´-TGACCTTCAGGAGAACGAGA-3´<br>R 5´-TCTTCTTCATGGCTCTGCGT-3
**SLC12A1**	F 5´-AACTTTGGGCCACGCTTCAC-3´<br>R 5´-CCACACAGGCCCCTACACAA-3
**SLC2A1**	F 5´-TTGCAGGCTTCTCCAACTGGAC-3´<br>R 5´-CAGAACCAGGAGCACAGTGAAG-3
**SLC22A1**	F 5´-CGCACCTTCATCCTGATGTACC-3´<br>R 5´-GAGCGGAGTAAAGGAAATCCAGG-3
**GLA**	F 5´-GACTGGGGAGTAGATCTGCTAAA-3´<br>R 5´-AGGAGAGCTTTGGCTTGAGG-3
**ECAD**	F 5´-TTGCCCAGAAAATGAAAAAGG-3´<br>R 5´-GTGTATGTGGCAATGCGTTC-3

**VEGF-A**	F 5´-GCACCCATGGCAGAAGGAG-3´<br>R 5´-TCCACCAGGGTCTCGATTG-3
**Vimentin**	F 5´-GACGCCATCAACACCGAGTT-3´<br>R 5´-CTTTGTCGTTGGTTAGCTGGT-3
**TGF-β**	F 5´-AAGCGGTCTTGCCCATCTTC-3´<br>R 5´-CTATGAGCAATGGCTGGCTTT-3
**α-SMA**	F 5´-AAAAGACAGCTACGTGGGTGA-3´<br>R 5´-GCCATGTTCTATCGGGTACTTC-3

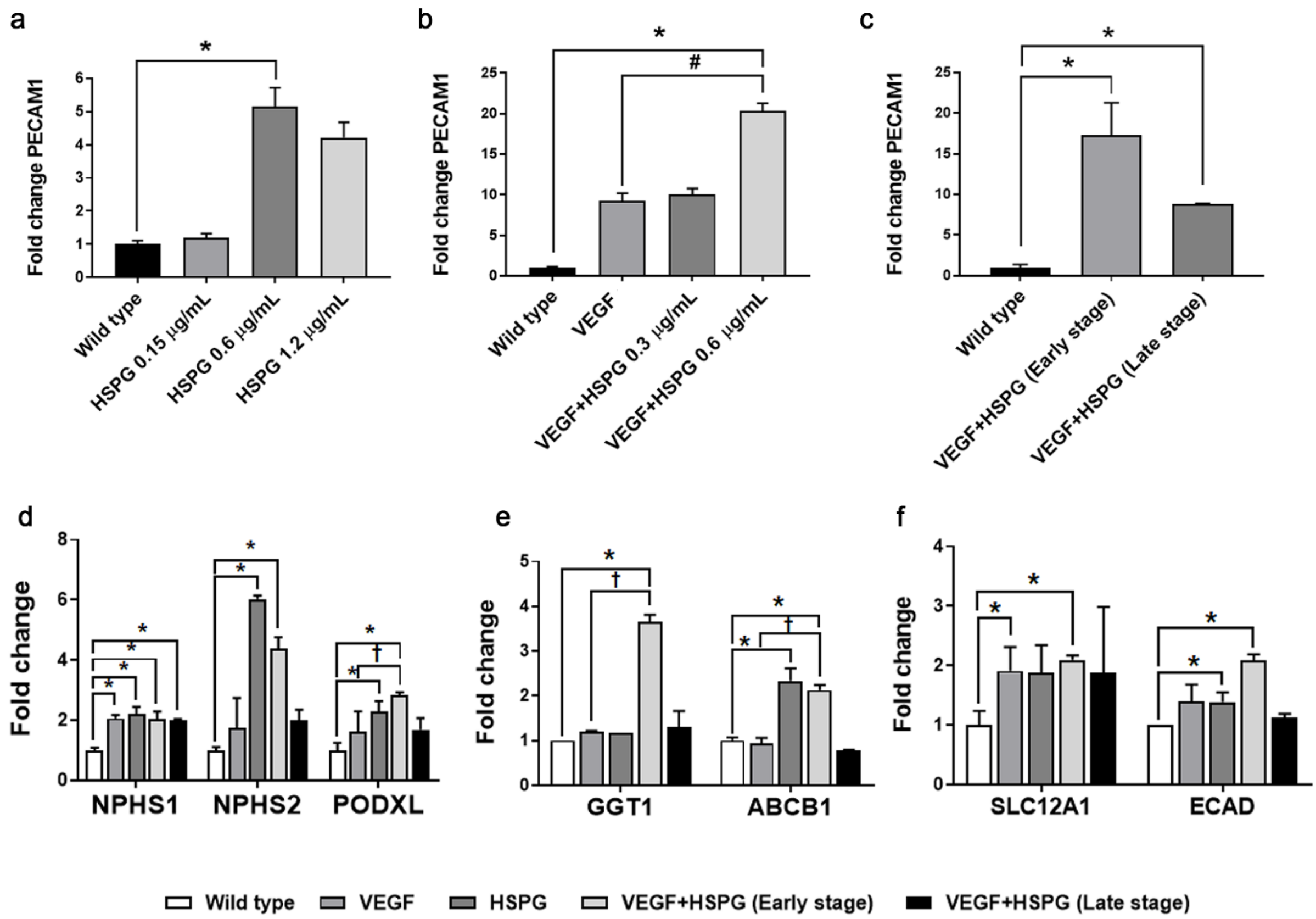

Supplementary Fig. 1. The combination treatment of HSPG and VEGF enhanced the gene expression of endothelial cells, podocytes, proximal tubule cells, distal convoluted tubular cells, and transporters of human iPSCs-derived kidney organoids

a,b qRT-PCR analysis of PECAM1 mRNA expression by HSPG alone, and VEGF co-treatment. **c** qRT-PCR analysis of PECAM1 mRNA expression at early and late stages by HSPG treatment. Values are mean ± SEM. *, vs. Wild type; #, vs. VEGF by the post hoc Mann-Whitney *U* test. **d-f** qRT-PCR analysis of NPHS1, NPHS2, and PODXL (podocyte genes), ABCB1 and GGT1 (drug transporter genes), SLC12A1 and ECAD (tubular epithelial transporter genes) at early and late stages during the period of differentiation by HSPG alone, and VEGF co-treatment. Values are mean ± SEM. *, vs. Wild type; #, vs. HSPG; †, vs. VEGF by the post hoc Mann-Whitney *U* test.

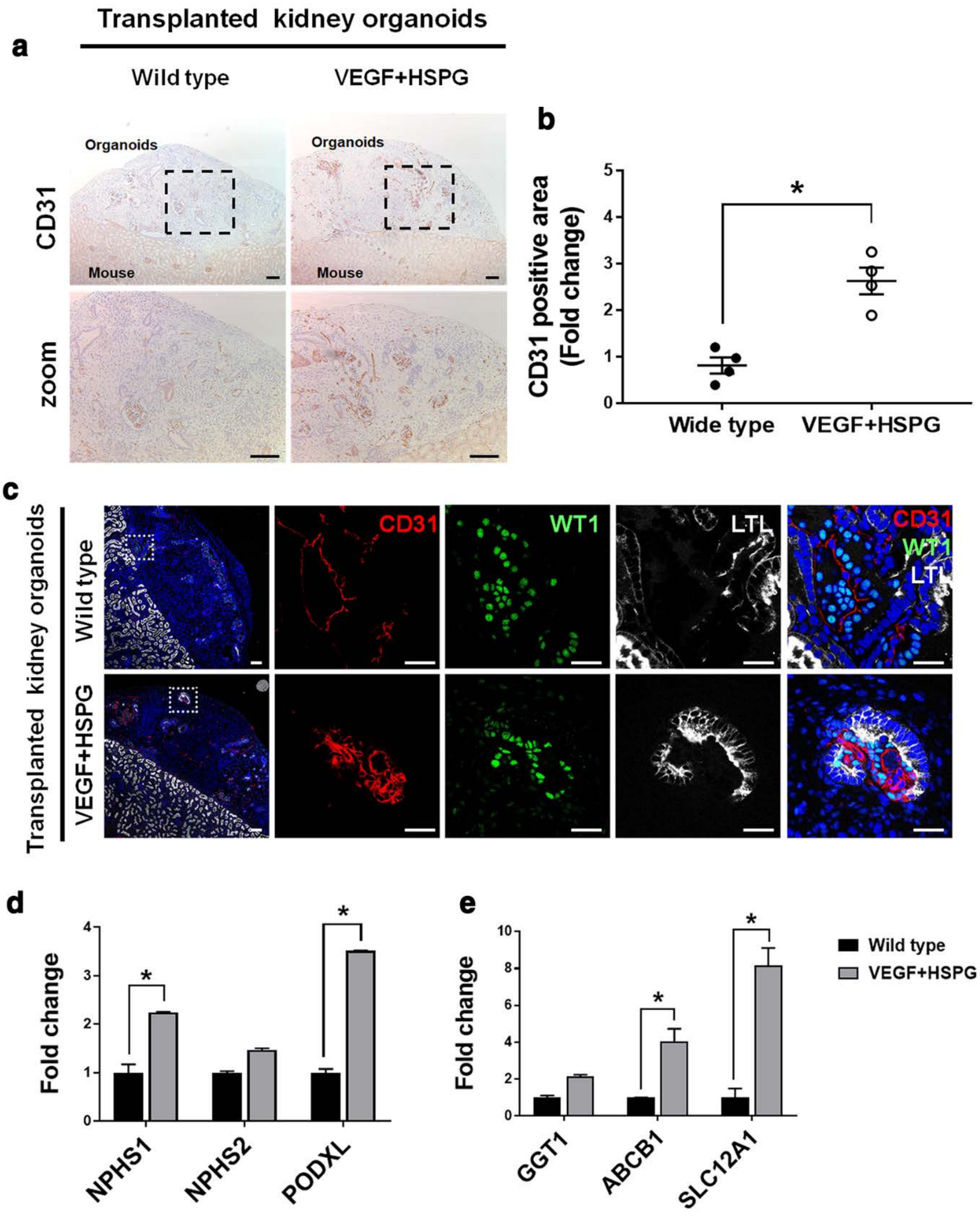
a
Transplanted kidney organoids
Wild type
VEGF+HSPG
Organoids
Mouse
CD31
zoom
b
CD31 positive area (Fold change)
Wide type
VEGF+HSPG
c
Transplanted kidney organoids
Wild type
VEGF+HSPG
CD31
WT1
LTL
d
Fold change
NPHS1
NPHS2
PODXL
e
Fold change
GGT1
ABCB1
SLC12A1
Wild type
VEGF+HSPG

Supplementary Fig. 2. Acceleration of the vascularization and gene expression of kidney cells in matured kidney organoids treated by HSPG and VEGF after transplantation beneath the renal capsule of NOD-SCID mouse

a,b Representative images of immunohistochemical staining with CD31 after transplantation with kidney organoid treated by HSPG and VEGF co-treatment. Scale bar = 500 μm. Quantification of CD31-positive area (%). **c** Representative confocal images of WT1 (podocyte), LTL (proximal tubule), and CD31 (vascular network) in vivo when transplanted with kidney organoid treated by HSPG and VEGF co-treatment. Scale bar = 50 μm. **d,e** qRT-PCR analysis of NPHS1, NPHS2, and PODXL (podocyte), GGT1 (proximal tubule gene), ABCB1 (drug transporter genes), SLC12A1(tubular epithelia transporter genes) (n=4). Values are mean ± SEM. *, vs. Wild type by the post hoc Mann-Whitney U test.

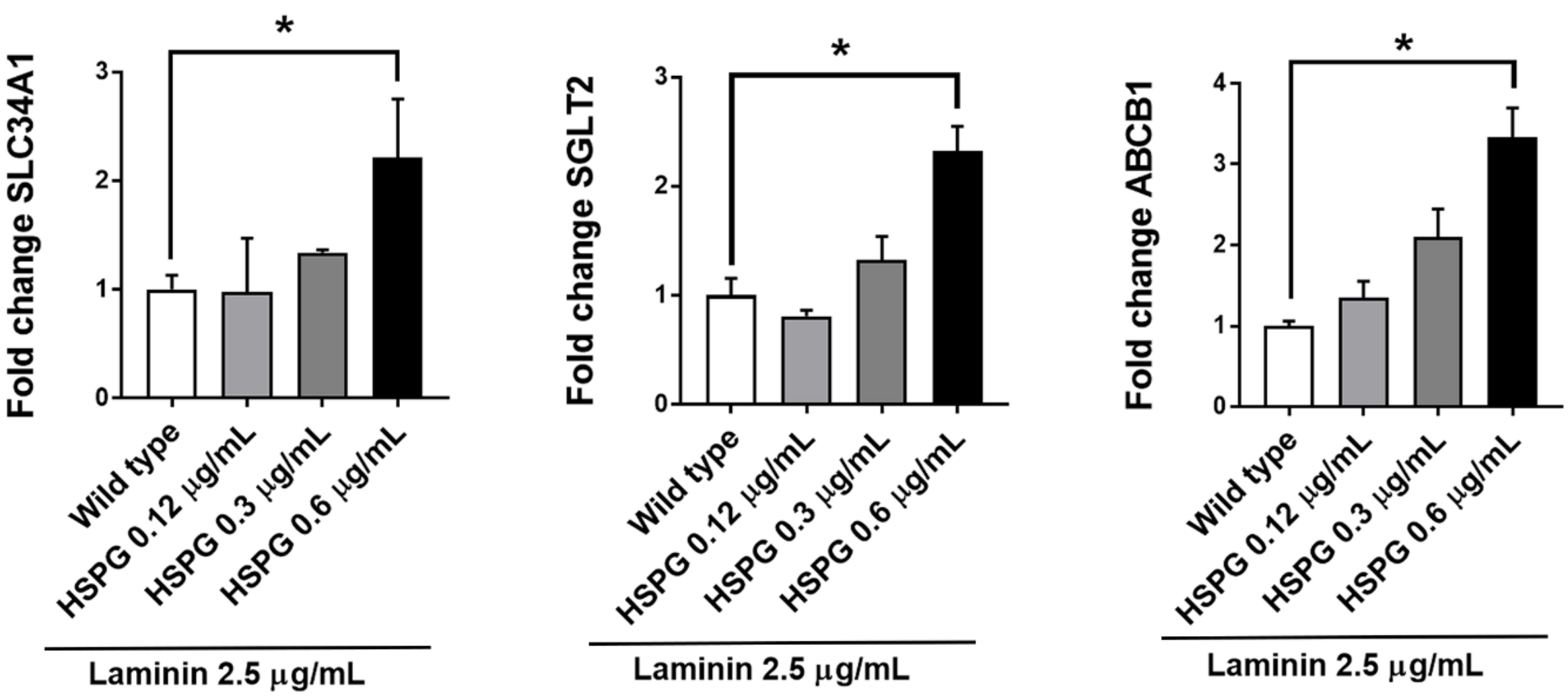

Supplementary Fig. 3. Increased gene expression of transporters of mKOPTCs cultured on wells coated with HSPG and laminin.

qRT-PCR analysis of SLC34A1, SGLT2 (solute transporter genes), ABCB1 (drug transporter genes on pre-coated HSPG with laminin. (four independent experiments, n≥3 well per condition). Values are mean ± SEM. *, vs. Wild type (Laminin only) by the post hoc Mann-Whitney *U* test.

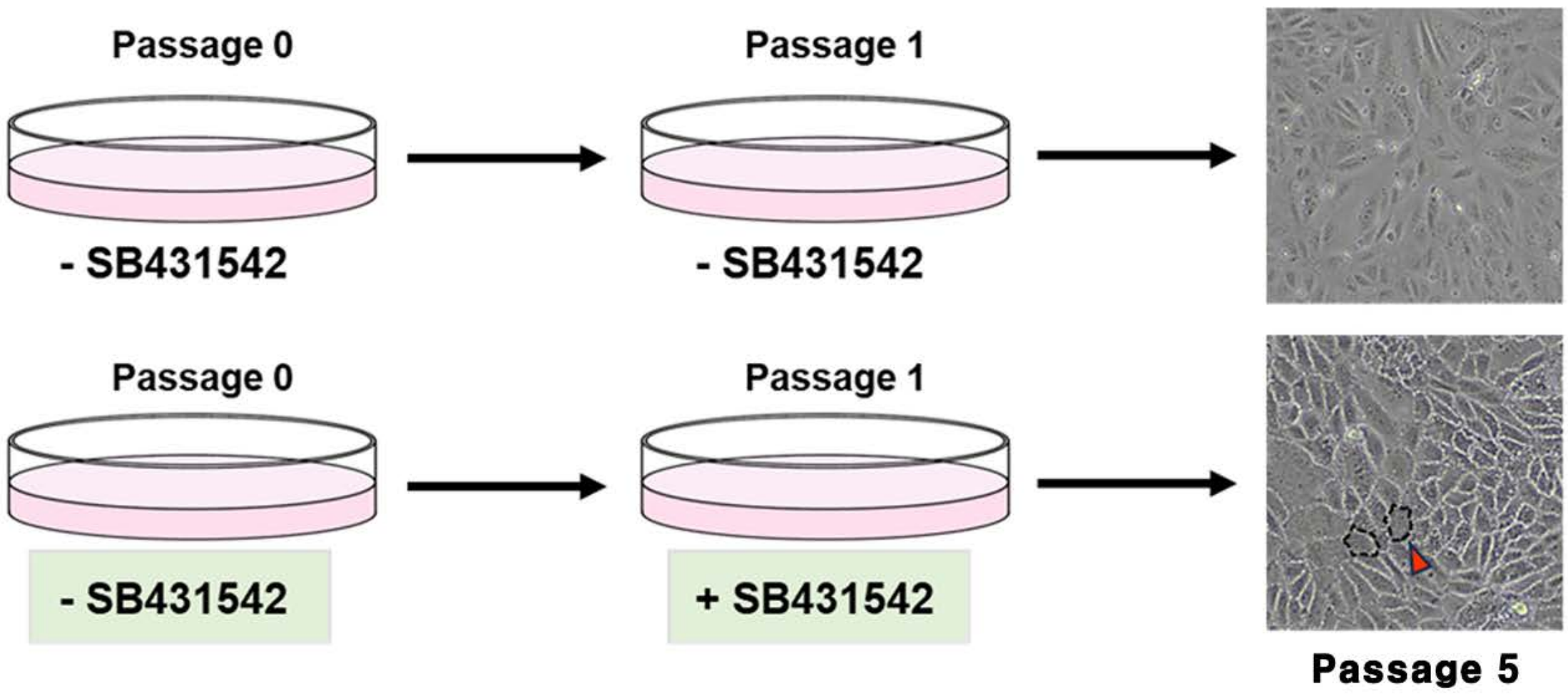

Supplementary Fig. 4. Improved morphology of mKOPTCs cultured with SB431542, as TGF-β inhibitor.

2D culture of mKOPTCs on Laminin+HSPG coated substrates with or without SB431542, as TGF-β inhibitor. On passage 5, mKOPTCs treated with 10 μM SB431542 exhibit cuboidal cell morphology during prolonged cell culture.

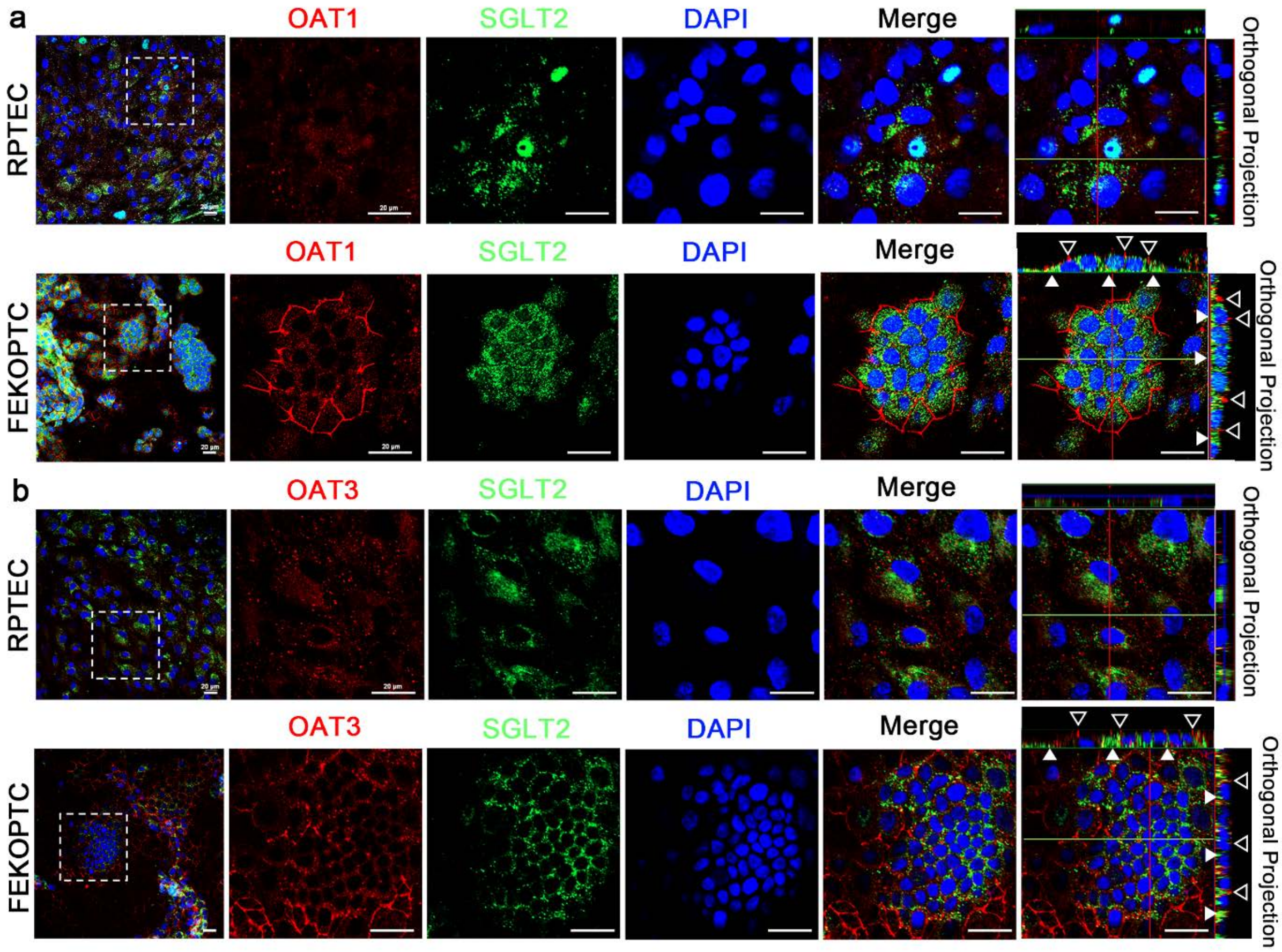

Supplementary Fig. 5. Representative immunofluorescence images of OAT1, OAT3, and SGLT2 in RPTEC and FEKOPTC.

Immunofluorescence confocal images showing 3D tubular structures in a Transwell system™. The red and green lines indicate the area of the yz-orthogonal projection. Immunofluorescence staining reveals the apical membrane of SGLT2 (glucose transporter) and the basolateral membranes of OAT1 (a) and OAT3 (b) (Organic anion transporters 1 and 3). Open arrowheads indicate OAT1 in the basolateral space. White arrowheads indicate SGLT2 in the apical space. Scale bar = 20 μm.

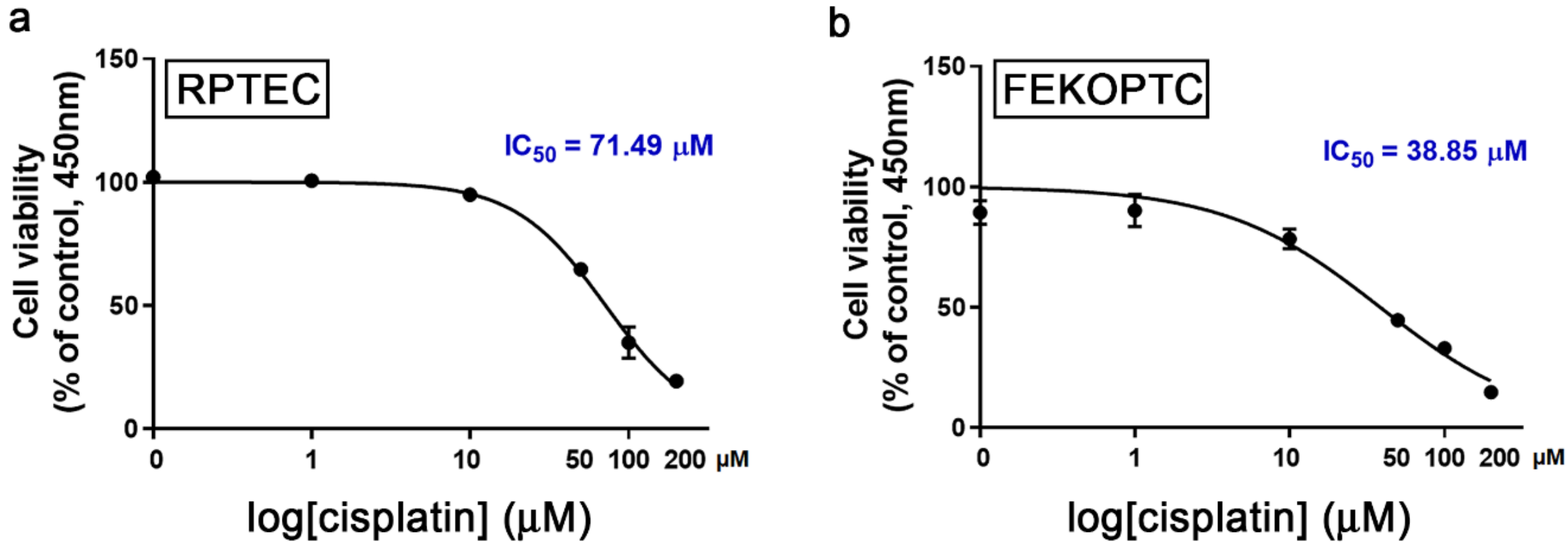

Supplementary Fig. 6. Nephrotoxicity evaluation of cisplatin in RPTEC and FEKOPTCs.

Cell viability was measured with the CCK-8 assay on RPTEC (a) and FEKOPTC (b) after exposure to cisplatin at 0, 1, 10, 50, 100, 200 μM for 24 h in serum-free medium. The concentration at which 50% of the cells are dead is shown as the IC50 value in the graphs. (Three independent experiments, n≥3 wells per condition). Values are mean ± SD.

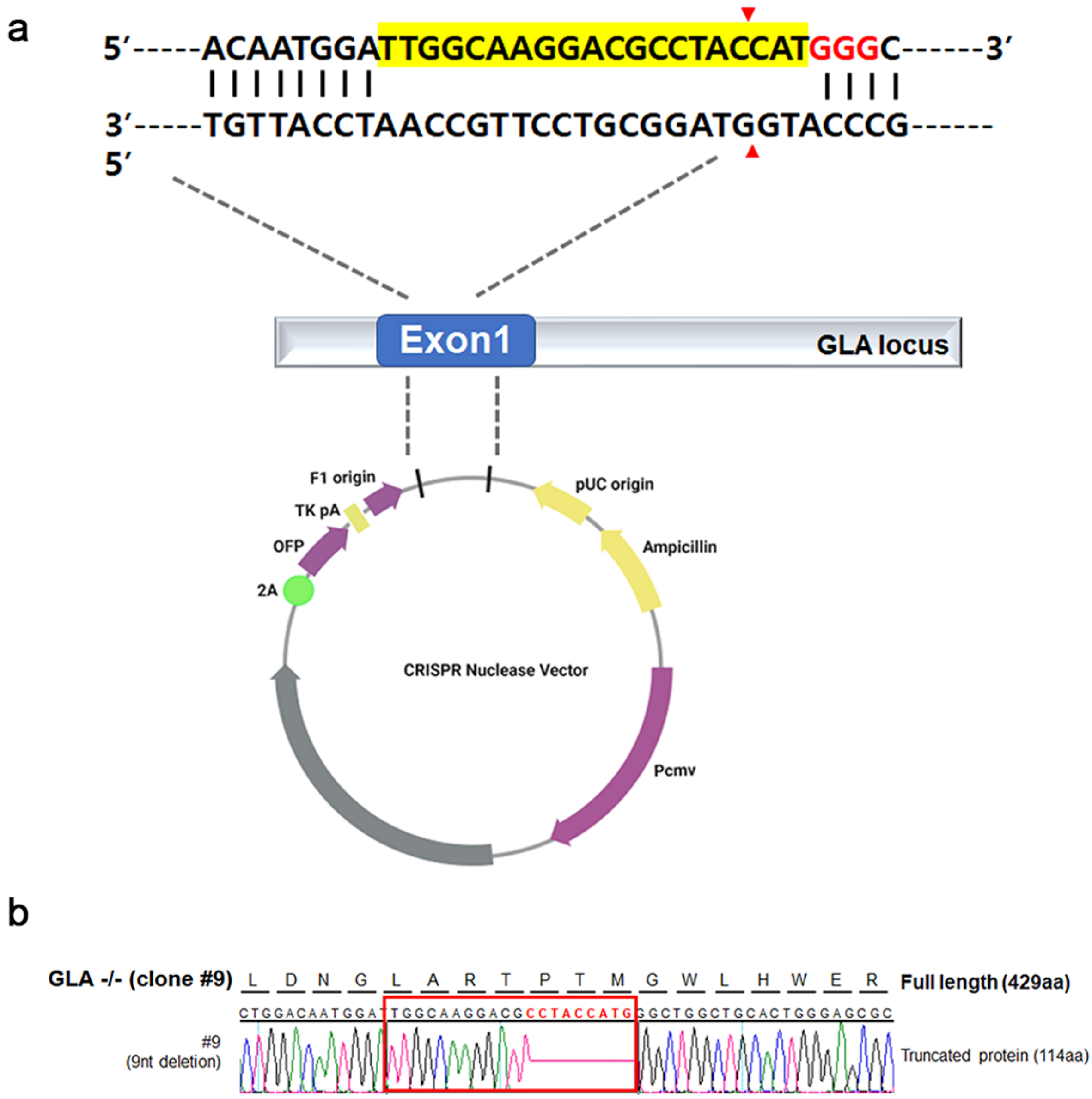

Supplementary Fig. 7. GLA knockout human iPSCs generated using CRISPR-Cas9.

a Schematic diagram of the single-guide RNA targeting site located exon1 of GLA gene. **b** Clones #9 Sequence after the knockout GLA target gene.
